# Manual Acupuncture or Combination with Vitamin B to Treat Diabetic Peripheral Neuropathy: A Systematic Review and Meta-Analysis of Randomized Controlled Trials

**DOI:** 10.1155/2020/4809125

**Published:** 2020-11-21

**Authors:** Hai lun Jiang, Peng Jia, Yi hua Fan, Meng dan Li, Can can Cao, Yuan Li, Yu zheng Du

**Affiliations:** ^1^First Teaching Hospital of Tianjin University of Traditional Chinese Medicine, Tianjin 300193, China; ^2^Tianjin University of Traditional Chinese Medicine, Tianjin 301617, China; ^3^National Clinical Research Center for Chinese Medicine Acupuncture and Moxibustion, Tianjin 300381, China; ^4^Tianjin Hospital, Tianjin 300221, China

## Abstract

**Methods:**

Randomized controlled trials on manual acupuncture treatment of DPN were retrieved from the Medline, Web of Science, PubMed, Cochrane Library, EMBASE, CNKI, WanFang, and VIP databases. Extracted research data were summarized in the tables, and methodological assessment was performed using the risk-of-bias assessment tool of Cochrane. Meta-analysis was performed by Revman 5.3, Stata 14.0, and TSA 0.9.5.10 Beta software.

**Results:**

A total of 18 randomized clinical trials (RCTs) were recruited: (1) 11 RCTs were acupuncture alone compared with vitamin B; (2) 7 RCTs were acupuncture combined with vitamin B compared with vitamin B, involving 1200 participants. Acupuncture alone improved clinical efficacy (*P* < 0.05) and nerve conduction velocity of the four peripheral nerves: peroneal nerve, tibial nerve, median nerve, and ulnar nerve (*P* < 0.05), but there was no significant difference between the group of acupuncture alone and the group of vitamin B (*P* = 0.36 > 0.05) in improving median nerve SCV (sensory nerve conduction velocity). Acupuncture combined with vitamin B improved clinical efficacy and nerve conduction velocity of the three peripheral nerves, peroneal nerve, tibial nerve, and median nerve (*P* < 0.05), and decreased the scores of the Toronto clinical scoring system (TCSS) (*P* < 0.05).

**Conclusion:**

Acupuncture alone and vitamin B combined with acupuncture are more effective in treating DPN compared to vitamin B. However, more high-quality RCTs on vitamin B combined with acupuncture are required to confirm our results.

## 1. Introduction

Diabetic peripheral neuropathy (DPN) has been described as “signs and symptoms of peripheral nerve dysfunction in patients with diabetes mellitus (DM) after the exclusion of other causes” [1]. Physical pain, numbness, decreased sensation, or other abnormal sensations are the main symptoms. Chronic distal symmetric polyneuropathy (DSPN) is the most common diabetic neuropathy [1]. An epidemiological study found that DPN prevalence is 7% for patients with type 1 diabetes and 22% for type 2 diabetes [2]. Most patients who were diagnosed with DPN present with pain, but a considerable number of patients without pain as a symptom were misdiagnosed and therefore did not receive adequate and timely treatment [3]. Mecobalamin is recommended for the treatment of DPN in patients with type 2 DM according to the China Food and Drug Administration guideline [4]. Although duloxetine and pregabalin are still the drug of choice (DOC) for treating painful diabetic neuropathy (PDN), adverse drug reactions in the cardiovascular system and digestive system have often been reported with significant safety concerns [5].

The prevention and management of DPN currently focus primarily on glucose control [6], while the signs and symptoms of DPN are always overlooked. In China, acupuncture is regarded as a “relatively safe” therapy, which is widely accepted with a long history, good reliability, feasibility, and ease of operation [7]. It was frequently used for treating conditions in the nervous system to avoid the severe side effects of chemical drugs.

Nerve conduction velocity is the gold standard to diagnose DPN [8], and TCSS is an effective tool for screening DPN with three domains including the neurological symptom score, neurological reflex score, and sensory function score to assess the DPN patient's symptoms and/or signs. TCSS is highly consistent with the neuroelectrophysiological function where lower scores indicate greater relief [9, 10].

DPN is considered by traditional Chinese medicine as one of “Bi Zheng (arthralgia syndrome)” or “Wei zheng (flaccidity syndrome)” under a larger category of “Xiao Ke (consumptive disease).” DPN is caused by blockage of “Qi and Xue (Qi and blood)” from perfusing limbs and muscles; then, it impedes the nourishment of meridians [11]. At this point, acupuncture can be applied to adjust the “Qi and Xue (Qi and blood)” of the human body and dredge the meridians [12]. So far, the RCTs on acupuncture treatment of DPN are poorly designed, with a generally poor methodological quality. What is more, no randomized controlled clinical trial from a multicenter has been published. Only two systematic reviews have been published, one of which analyzed articles until April 2013 while the other review analyzed articles up to June 2017 [13, 14]. However, neither article has further in-depth analysis of heterogeneous indicators. Some limitations of these two studies include the following: (1) not all of the included articles were RCTs; (2) most of the participants had diabetic peripheral neuropathic pain (DPNP) and hence could not represent most DPN; and (3) Chinese databases were not searched [12]. In this review, we solely used RCT data to evaluate the efficacy of acupuncture treatment on DPN. We compared the application of combining acupuncture and vitamin B with vitamin B or compared acupuncture alone with vitamin B in treating DPN, respectively.

## 2. Materials and Methods

### 2.1. Study Registration

This protocol of systematic review and meta-analysis has been drafted under the guidance of the Preferred Reporting Items for Systematic Reviews and Meta-Analyses protocols (PRISMA-P). Moreover, it has been registered on the open science framework (OSF) on May 14, 2020 (registration number: DOI 10.17605/OSF.IO/PZ5GC).

### 2.2. Search Strategy

Keywords such as “acupuncture,” “diabetic peripheral neuropathy,” and “DPN” were used to search against databases including Medline, Web of Science, PubMed, Cochrane Library, EMBASE, CNKI, WanFang, and VIP, for RCTs, systematic reviews, or meta-analyses published by April 5, 2020. There were no language restrictions. The two investigators independently reviewed all literature to determine its inclusion (Hai lun Jiang and Peng Jia). Disagreements were adjudicated by a third investigator (Yi hua Fan). The meta-analysis was conducted following the guidelines of Preferred Reporting Items for Systematic Reviews and Meta-Analyses (PRISMA). The search strategy of EMBASE and CNKI is listed in Table S1, and PubMed search history is listed in Table S3

### 2.3. Inclusion Criteria

1. Patients with diabetic peripheral neuropathy.
2. RCT studies comparing manual acupuncture with vitamin B in the treatment of DPN; the manual acupuncture study group includes both acupuncture alone and acupuncture coupled with vitamin B; the control group was treated only with chemical drugs, and vitamin B must be included.
3. Primary outcome: (1) clinical efficacy. Secondary outcomes: (1) motor nerve conduction velocity (MCV) of the peroneal nerve; (2) sensory nerve conduction velocity (SCV) of the peroneal nerve; (3) motor nerve conduction velocity (MCV) of the tibial nerve; (4) sensory nerve conduction velocity (SCV) of the tibial nerve; (5) motor nerve conduction velocity (MCV) of the median nerve; (6) sensory nerve conduction velocity (SCV) of the median nerve; (7) motor nerve conduction velocity (MCV) of the ulnar nerve; and (8) Toronto clinical scoring system (TCSS). Supplementary explanation: the definition of clinical efficacy was not similar among trials. The included trials' clinical efficacy evaluation was based on the following criteria [15]:
4. *Effectiveness*. Symptoms and/or signs of peripheral nerve dysfunction improved, and MCV or SCV increased.
5. *Ineffectiveness*. Symptoms and/or signs of peripheral nerve dysfunction had not improved, or MCV or SCV did not obviously improve.

When the effect of symptoms and/or signs is inconsistent with the effect of nerve conduction velocity, the lower effective parameter is applied to show the comprehensive effect.

### 2.4. Exclusion Criteria

1. Duplicated published articles
2. Nonhuman limb acupoints
3. Incomplete data from the articles
4. The trials were rated as low quality by Zhao et al.'s criteria [16]

### 2.5. Data Extraction

Two independent investigators (Hai lun Jiang and Peng Jia) separately extract data, involving the name of authors, year of publication, sample size, age of participants, duration of DPN, intervention measures, intervention time, outcome indicators, reinforcing and reducing, acupoints, and needle retaining time. When disagreements occurred, two independent investigators (Hai lun Jiang and Peng Jia) discussed to resolve the issues. If disagreements persisted, then the third investigator (Yi hua Fan) was consulted to make the final decision. Additional information was obtained by contacting authors directly via call or email.

### 2.6. Risk-of-Bias Assessments

Two independent reviewers (Hai lun Jiang and Peng Jia) evaluated the quality of the included RCTs separately, based on the Cochrane risk-of-bias criteria [17]. When disagreements occurred, two independent investigators (Hai lun Jiang and Peng Jia) discussed to resolve the issues. If disagreements persisted, then the third investigator (Yi hua Fan) was consulted to make the final decision.

### 2.7. Data Synthesis and Statistical Analysis

In this meta-analysis, the Revman 5.3 (developed by the UK's International Cochrane Collaboration) and Stata 14.0 (developed by the USA's StataCorp LLC) software were used for analysis. Relative risks (RR) were used to express dichotomous variables, whereas the mean difference (MD) and 95% confidence interval (CI) were used to denote continuous variables. The chi-squared tests were used to assess statistical heterogeneity. When *I*^2^ < 50% or chi-squared test *P* ≥ 0.1, a fixed-effect model was applied. The source of heterogeneity was analyzed, when *I*^2^ > 50% or chi-squared test *P* < 0.1. In the absence of clinical heterogeneity or methodological heterogeneity, the random-effect model was applied. Statistical significance was set at *P* < 0.05. To test publication bias, Egger's test was performed. Moreover, a sensitivity analysis was conducted to test the stability of the results.

Besides, trial sequence analysis using TSA 0.9.5.10 Beta (developed by the Copenhagen Trial Unit's Centre for Clinical Intervention Research) was used to calculate the appropriate sample size for the meta-analysis and to assess the statistical boundaries for futility and efficacy. The*X*axis represents the sample size (participants),*Y*axis represents the statistic*Z*values, two symmetrical red curves represent the boundary value of trial sequence analysis (TSA boundary value), and symmetrical red horizontal dashed lines represent the conventional boundary values (*Z* = 1.96,*P* = 0.05(two-sided)). If the cumulative*Z*value does not cross the TSA boundary value or RIS, it indicates that the sample size is insufficient, to recommend continuing the series of experiments. If the cumulative *Z* value exceeds both the TSA boundary value and the RIS, then the sample size is sufficient. If the cumulative *Z* value exceeds the TSA boundary value and does not reach the RIS, it means that a reliable conclusion can be drawn in advance even if the sample size is insufficient. According to the TSA method, when the cumulative *Z* value has crossed the RIS, or the cumulative *Z* value intersects the TSA boundary value, the series of tests can be recommended to stop (TSA parameter setting: type I error probability 5%, type II error probability of 20%, and the relative risk reduction (RRR = −15%), to estimate the required information size (RIS)) [18].

## 3. Results

**Figure 1:**
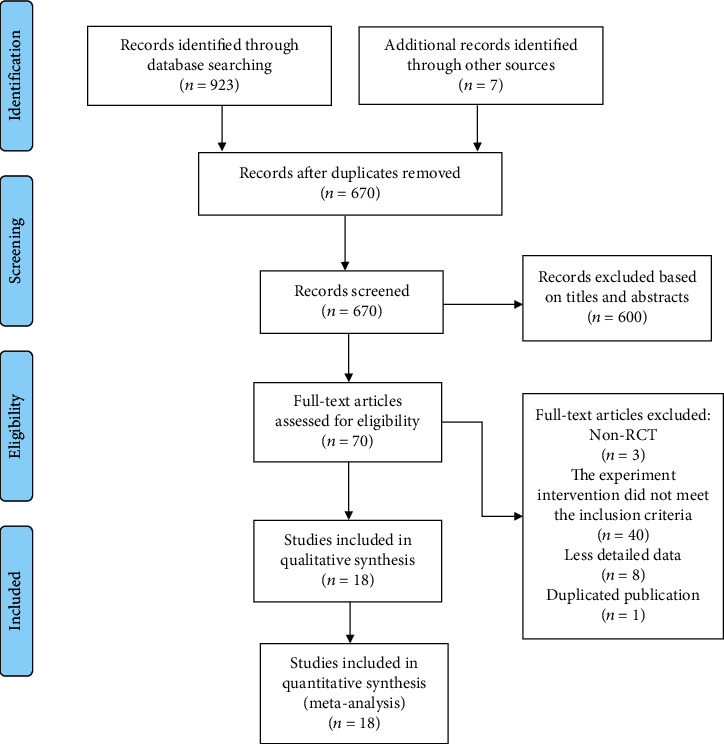
Flow chart of study identification and selection.

A total of 18 articles were selected, and the process is shown in Figure1 [19–36].

**Figure 2:**
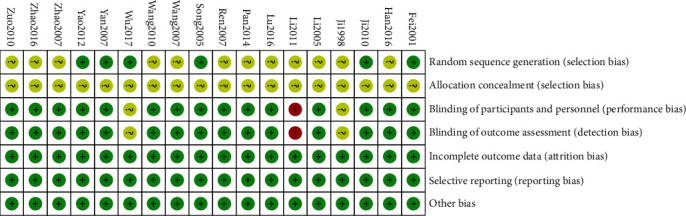
Risk-of-bias summary.

Only 7 of 18 randomized trials reported methods of randomization. No trial reported allocation concealment. Blinding does not affect the measure of nerve conduction velocity (NCV), and only one study reported blinding in this analysis. 15 trials showed low risk in blinding participants or outcome assessment. All studies were of low risk of incomplete outcome data, selective reporting, or other bias.  Figure 2 outlines the quality and risk-of-bias evaluation of the included studies. Risk-of-bias assessments are shown in Figure 2.

### 3.1. The Basic Characteristics of the Inclusion Study

Name of author, year of publication, sample size, age of participants, duration of DPN, intervention measures, intervention time, and outcome indicators are summarized in Table 1, and acupoints, reinforcing and reducing, and needle retaining time are summarized in Table 2.

### 3.2. Meta-Analysis Result

In the experimental group, “acupuncture alone” means “the intervention measure contains manual acupuncture, but without neurotrophic drugs” and “acupuncture+vitamin B” means “the intervention measure contains manual acupuncture and vitamin B, but no other neurotrophic drugs.”

In the control group, “vitamin B” means “the intervention measure contains vitamin B as the only neurotrophic drug.”

#### 3.2.1. Description of Statistical Results

Out of the 18 trials retrieved from searching published RCTs and systematic reviews, 11 trials were acupuncture alone vs. vitamin B involving 699 participants (there are 356 participants in the acupuncture alone group and 343 participants in the vitamin B group) and 7 trials were acupuncture combined with vitamin B vs. vitamin B involving 501 participants (there are 266 participants in the acupuncture+vitamin B group and 235 participants in the vitamin B group).

#### 3.2.2. Acupuncture Alone vs. Vitamin B (Figure 3)

**Figure 3:**
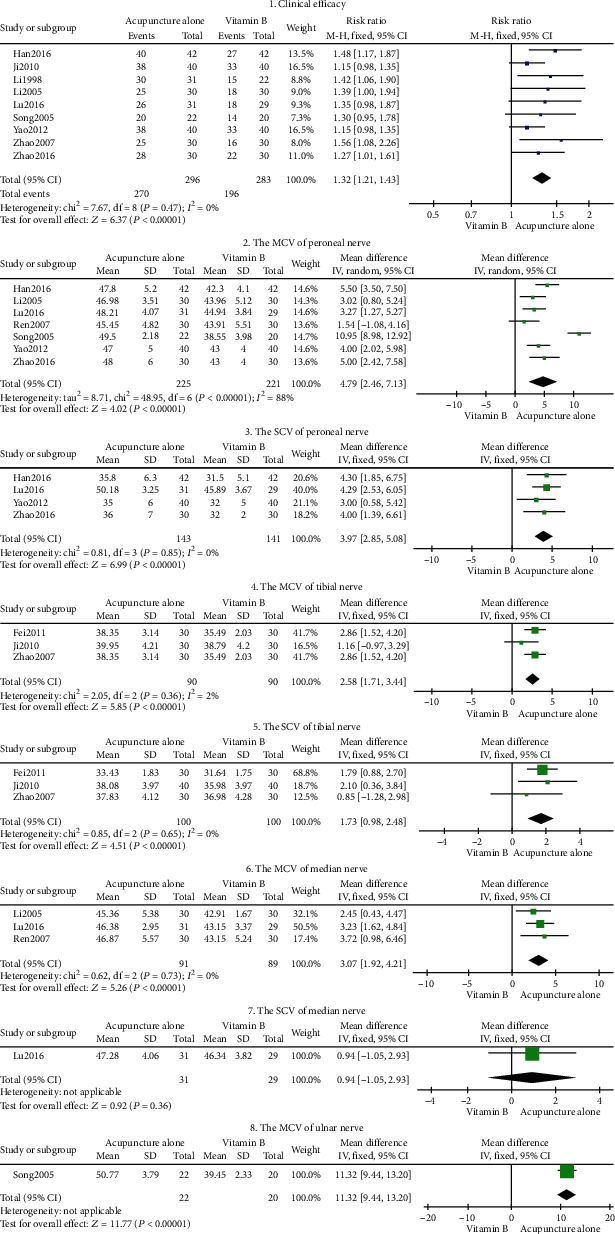
Forest plot of acupuncture alone.

Eight outcome indicators were used to compare acupuncture alone to vitamin B. The pooled results exhibited significant differences in clinical efficacy, peroneal nerve MCV, peroneal nerve SCV, tibial nerve MCV, tibial nerve SCV, median nerve MCV, and ulnar nerve MCV (*P* < 0.05), respectively. As shown in Figure 3, our findings suggest that acupuncture alone is more effective than using vitamin B in DPN (*P* < 0.05) therapy. There was no significant difference between the SCV of the median nerve of the acupuncture alone group and that of the vitamin B group (MD = 3.07, 95% CI: 1.92-4.21, *P* = 0.36 > 0.05; tag 7 in Figure 3). In this RCT, the efficacy of vitamin B (lipoic acid) in the treatment of DPN was compared with that of acupuncture alone. Given that only one study was included in this outcome indicator, sensitivity analysis could not be carried out for further verification.

#### 3.2.3. Acupuncture+Vitamin B vs. Vitamin B (Figures 4 and 5)

**Figure 4:**
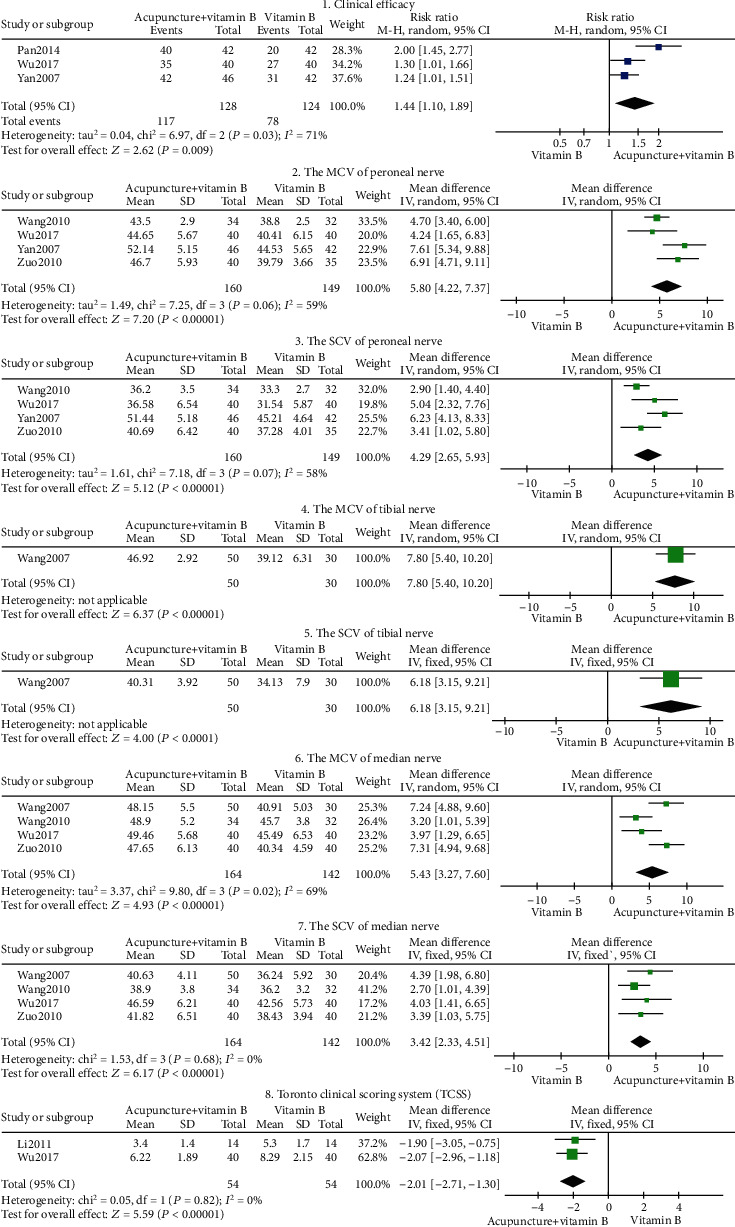
Forest plot of acupuncture+vitamin B.

Eight outcome indicators were used to compare acupuncture+vitamin B with vitamin B. The pooled results displayed significant differences in clinical efficacy, peroneal nerve MCV, peroneal nerve SCV, tibial nerve MCV, tibial nerve SCV, median nerve MCV, median nerve SCV, and Toronto clinical scoring system (*P* < 0.05). As shown in Figure 4, our findings suggest that acupuncture combined with vitamin B is better than using vitamin B alone, in the treatment of DPN.

**Figure 5:**
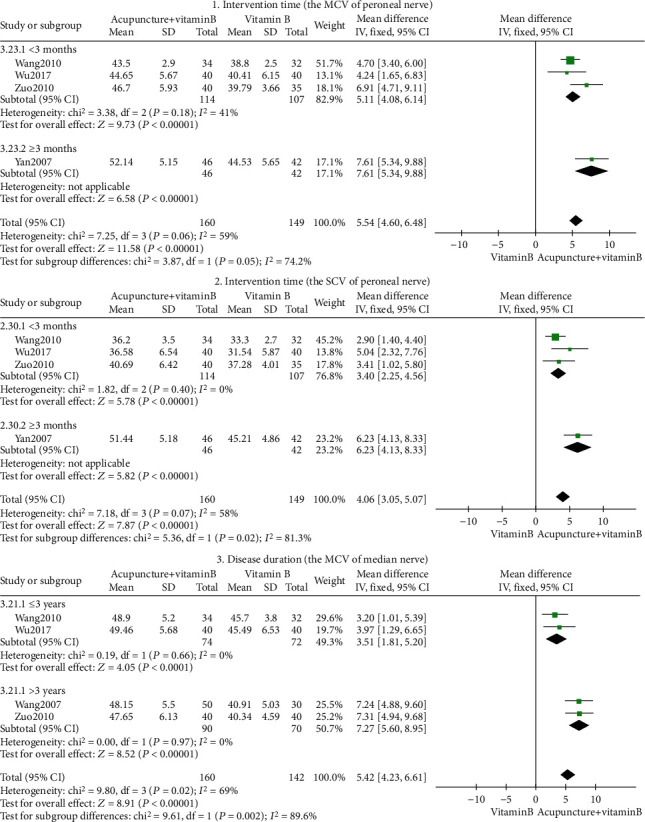
Acupuncture+vitamin B's subgroup analysis forest plot.

Peroneal nerve exposure's heterogeneity in MCV and SCV was high (*I*^2^ = 59%, *I*^2^ = 58%; Figure 4, tags 2 and 3), but all indexes were on the right of the invalid line. The MCV and SCV of the peroneal nerve had no significant clinical heterogeneity or methodological heterogeneity. We further conducted sensitivity analyses on MCV and SCV of the peroneal nerve, and the outcome was stable. Subgroup analysis was conducted based on intervention time (<3 months, ≥3months), indicating that heterogeneity was derived from intervention time (Figure 5, tags 1 and 2). Both short- and long-term acupuncture treatment therapy enhanced peroneal nerve MCV and peroneal nerve SCV (*P* < 0.05). While some heterogeneity was present (Figure 5, tag 1), it was still within acceptable limits. Given that treatment time is associated with restoration of nerve conduction velocity, there is still a need to determine the long-term effectiveness of acupuncture as DPN often recur after treatment [37, 38].

The heterogeneity in the MCV of the median nerve was high (*I*^2^ = 69%; tag 6 in Figure 4). Subgroup analysis conducted based on disease duration (≤3 years, >3 years) revealed heterogeneity in disease duration (Figure 5, tag 3). Acupuncture intervention can improve the MCV of the median nerve for both short-time and long-time disease duration (*P* < 0.05).

#### 3.2.4. Subgroup Analysis (Figure 6)

**Figure 6:**
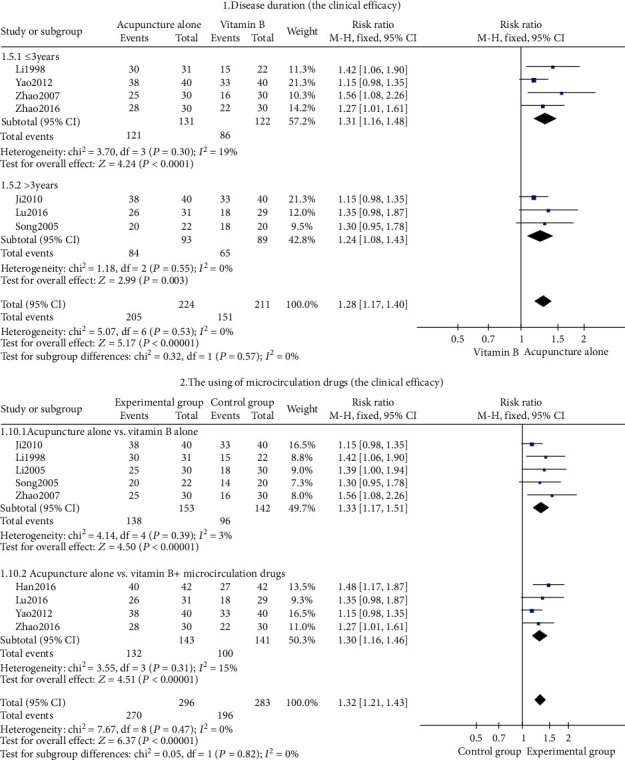
The clinical efficacy's subgroup analysis forest plot.

In the study of clinical efficacy, subgroup analysis was conducted based on the duration of the disease (≤3 years and >3 years) (Figure 6, tag 1). The result showed that acupuncture alone in the short- or long-term duration of the disease was better than using vitamin B (*P* < 0.05). We further conducted another subgroup analysis based on drugs used in the control group (with microcirculation drugs and no microcirculation drugs) (Figure 6, tag 2). The result showed that acupuncture alone was better than using vitamin B or vitamin B combined with microcirculation drugs (*P* < 0.05).

### 3.3. Trial Sequence Analysis (Figures 7 and 8)

**Figure 7:**
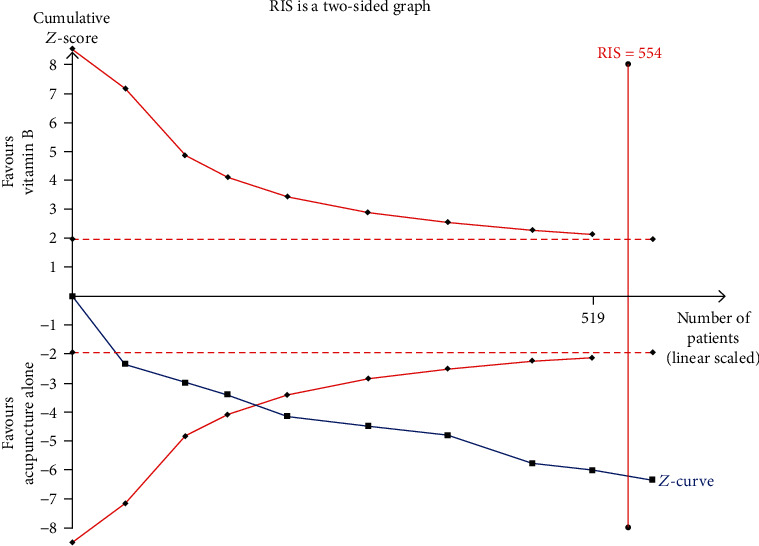
Trial sequence analysis of acupuncture alone (the clinical efficacy).

The TSA of acupuncture alone revealed that the cumulative *Z*-curve crossed the conventional boundary value (*Z* = 1.96, *P* = 0.05(two-sided)) and TSA boundary value and met the RIS (554 cases) (Figure 7). This result means that the cumulative sample size meets expectations; no more trials were needed.

**Figure 8:**
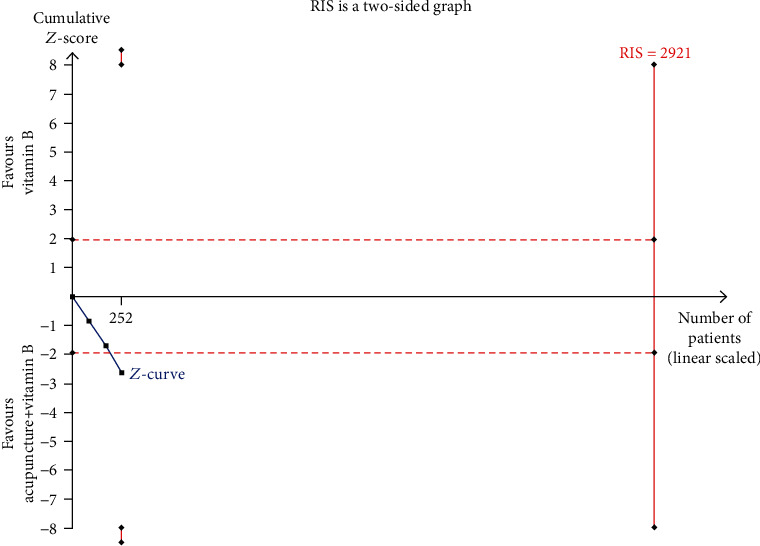
Trial sequence analysis of acupuncture+vitamin B (the clinical efficacy).

The TSA of acupuncture combined with vitamin B revealed that the cumulative *Z*-curve crossed the conventional boundary value (*Z* = 1.96, *P* = 0.05 (two-sided)) but did not reach the TSA monitoring boundary and RIS (2921 cases) (Figure 8). This means that the cumulative sample size did not meet expectations; more trials were needed.

### 3.4. Safety Analysis

Two RCTs [27, 36] reported no adverse event, and no mention was made of the others.

### 3.5. Publication Bias

Egger's test was performed to evaluate the publication bias of the primary outcome. Nine studies were evaluated for the clinical efficacy of acupuncture alone (Egger's test: *P* = 0.005), and the results showed a statistically significant difference. Besides, the statistical significance of Egger's test indicates publication bias. Three studies were evaluated for the clinical efficacy of acupuncture combined with vitamin B (Egger's test: *P* = 0.207), and the results revealed no publication bias.

### 3.6. Sensitivity Analysis

Sensitivity analysis was performed to test the stability of the results, and the results showed that all indicators were stable.

## 4. Discussion

DPN pathogenesis involves interactions of multiple factors; hence, it is not yet clear. Since hyperglycemia is a well-known factor affecting many internal metabolic pathways, the key approach to DPN treatment is therefore strict control of hyperglycemia. Additionally, abnormal lipid metabolism, insulin resistance, and neurotrophasthenia may also participate in the metabolic pathway [39, 40]. Peripheral nerve edema, demyelination, and axonal degeneration are associated with the deposition of catabolic blood glucose products on peripheral nerves [41, 42].

Vitamin B restores neurotrophic factors and repairs nerves, which is used as supplementary treatment of axonal cell damage and nerve demyelination changes. Mecobalamin, a form of vitamin B_12_ with a high neuronal affinity, was shown to be able to repair the damaged myelin sheath and promote axon regeneration [43]. However, the efficacy of mecobalamin alone is often unsatisfactory in the treatment of DPN [44]. Combination of multiple vitamin B [27, 28] has a protective effect on micrangium of diabetic patients [45] and alleviates the symptoms. The animal experiment demonstrated that vitamin B_1_ can prevent abnormal lipid metabolism [46]. Lipoic acid, a form of vitamin B-like compound, was a natural antioxidant and could improve nerve conduction velocity [47]. Compared with mecobalamin, lipoic acid was more effective in treating DPN [48]. In addition, the combined use of neurotrophy medicine and drugs that improve microcirculation, such as alprostadil, has demonstrated synergistic action (Figure 6, tag 3) in the treatment of type 2 DM [49] and thus is recommended by the Chinese guideline [4]. Dilation of the vessels to enhance microcirculation is an important treatment mechanism [50].

Acupuncture exhibits a good therapeutic effect on DPN due to its direct local effect on microcirculation and its role in electrophysiological activity [51]. What is more, acupuncture can effectively reduce plasma neuropeptide Y (NPY) [52], which is one of the main risk factors for the development of type 2 diabetes via constriction of blood vessels and changes in the metabolic environment of the body [53], especially on lipid metabolism [54]. Reducing plasma neuropeptide Y (NPY) effectively delays the occurrence of diabetes and its complications [55]. Furthermore, acupuncture protects islet cells, increases insulin sensitivity, and promotes the secretion of insulin in diabetic patients to regulate the patient's blood glucose and lipid [56].

Our subgroup analysis indicates that acupuncture can achieve clinical efficacy in a short course of treatment which shows a good curative effect on patients with duration of DPN over 3 years. Of all included studies, Zusanli (ST36), Sanyinjiao (SP6), Quchi (LI11), and Yanglingquan (GB34), which are mainly distributed in the forearm and foreleg, are frequently selected for stimulation. In 18 RCTs, their acupoint frequencies were 13, 12, 11, and 9, respectively. An examination found that the distal part of extremities is the most common lesion site [57]. It is consistent with the hypothesis that an “acupoint can be used to treat unusual symptoms around this acupoint” [58].

It is related to their local anatomy structure, as these acupoints are close to the dorsal cutaneous nerve of the forearm, lateral sural cutaneous nerve, radial nerve, peroneal nerve, deep peroneal nerve, tibial nerve, etc. directly. Furthermore, an animal experiment showed that stimulating Zusanli (ST36) improves blood glucose regulation [59].

The onset and development of DPN can be explained as deficiency in the root and excess in the branch, according to the basic theory of traditional Chinese medicine (TCM), deficiency of “Qi” and “Xue” is the root, and blood stasis and phlegm are the branches [60, 61]. Sanyinjiao (SP6) is the location where the liver meridian, spleen meridian, and kidney meridian meet and can therefore nourish these three zang-fu organs (kidney, liver, and spleen). Needling Sanyinjiao (SP6) also harmonize “Qi” and “Xue” and balance Yin-Yang. Simply put, needling these acupoints can dredge the meridian and promote Qi and blood circulation. Ultimately, it relieves pain, numbness, and other symptoms, to achieve a good effect of DPN therapy. The results suggested that DPN should be treated using acupuncture. And the result of trial sequence analysis shows that the sample size of RCTs on acupuncture alone to treat DPN was enough but acupuncture combined with vitamin B to treat DPN was not.

## 5. Limitations

1. Only one of the 18 included trials was blind research, which might affect the evaluation of clinical efficacy according to the *Cochrane Handbook*. But it has no effect on the measurement of nerve conduction velocity
2. The number of the 18 included studies was small (a total of 1200 participants were involved), and 17 trials were in the Chinese language that may lead to publication bias. Based on the result of trial sequence analysis, the sample size of RCTs on acupuncture combined with vitamin B to treat DPN was not enough
3. Although the 18 RCTs showed that acupuncture alone and acupuncture combined with vitamin B are more effective than vitamin B in the treatment of DPN, there are some variations in acupuncture that may cause different curative effects
4. None of the studies reported long-term effects and safety following acupuncture treatments
5. There is no uniform international standard for the definition of clinical efficacy; the clinical efficacy evaluation was based on guiding principles for clinical research of new Chinese medicine [15]

## 6. Conclusions

The main role of acupuncture compared with vitamin B is to improve clinical efficacy. Besides, acupuncture significantly increases nerve conduction velocity and decreases the scores of the Toronto clinical scoring system (TCSS). However, further multicenter studies with large samples and high-quality RCTs on acupuncture combined with vitamin B are required, to more reliably assess the effect of acupuncture on DPN. Meanwhile, it is necessary to set standard criteria for evaluating clinical effectiveness.

## Figures and Tables

**Table 1:** The characteristics of the included trials.

References	Sample size (T/C)	Age (year)	Disease duration (year)	Intervention		Intervention time (days)	Outcome
				Treatment	Control		
Fei 2011	30/30	T: 54 ± 1 C: 55 ± 1	T: 2.43 ± 2.23 C: 2.53 ± 0.34	Ac	Mec (p.o)	30	(4), (5)
Han 2016	42/42	T: 56.3 C: 56.2	/	Ac	Mec (p.o)+nimodipine (p.o)	56	(1), (2), (3)
Ji 2010	40/40	T: 60.7 ± 4.26 C: 62.2 ± 4.13	T: 3.77 ± 1.16 C: 3.44 ± 1.29	Ac	Mec (i.m)	28	(1), (4), (5)
Li 1998	31/22	/	T: 3 weeks–2 years C: 3 weeks–2 years	Ac	VitB_1_, B_12_ (i.m)	30	(1)
Li 2005	30/30	All: 56.1 ± 3.2	/	Ac	Mec (i.v)	120	(1), (2), (6)
Li 2011	14/14	/	/	Ac+Mec (p.o)	Mec (p.o)	45	(9)
Lu 2016	31/29	T: 66 ± 7 C: 64 + 7	T: 3.6 ± 1.3 C: 3.5 ± 1.1	Ac	Lipoic acid (i.v.drip)+alprostadil (i.v.drip)	30	(1), (2), (3), (6), (7)
Pan 2014	42/42	/	/	Ac+Mec (p.o)	Mec (p.o)	90	(1)
Ren 2007	30/30	T: 63.±12.58 C: 60.±11.47	T: 5.64 ± 4.77 C: 5.81 ± 4.38	Ac	VitB_1_, B_12_ (i.m)	30	(2), (6)
Song 2005	22/20	T: 58.9 ± 5.24 C: 58.9 ± 5.24	T: 6.91 ± 3.15 C: 6.33 ± 3.56	Ac	VitB_12_ (i.m)+VitB_1_, B_6_ (p.o)	30	(1), (2), (8)
Wang 2007	50/30	T: 55.8 C: 56.1	T: 6.5 C: 7	Ac+VitB	VitB	30	(4), (5), (6), (7)
Wang 2010	34/32	T: 56.1 ± 5.33 C: 58.4 ± 8.52	T: 2.91 ± 2.38 C: 2.86 ± 2.59	Ac+Mec (i.v)	Mec (i.v)	28	(2), (3), (6), (7)
Wu 2017	40/40	T: 53.4 ± 8.31 C: 52.9 ± 8.45	T: 2.62 ± 0.56 C: 2.52 ± 0.48	Ac+Mec (p.o)	Mec (p.o)	14	(1), (2), (3), (6), (7), (9)
Yan 2007	46/42	T: 46.7-74.98 C: 46.5-75.6	T: 0.8-12.4 C: 0.7-11.8	Ac+Mec (p.o)	Mec (p.o)	90	(1), (2), (3)
Yao 2012	40/40	T: 54.5 C: 53.4	T: 0.67 C: 0.58	Ac	Mec (p.o)+nimodipine (p.o)	28-56	(1), (2), (3)
Zhao 2007	30/30	T: 62.±7.33 C: 62.1 ± 7.93	T: 2.71 ± 2.58 C: 2.61 ± 2.22	Ac	Mec (p.o)	60	(1), (5)
Zhao 2016	30/30	All: 53 ± 9.2	All: 0.58 ± 0.25	Ac	Mec (p.o)+nimodipine (p.o)	56	(1), (2), (3)
Zuo 2010	40/35	T: 57.6 C: 57.1	T: 8.7 C: 8.5	Ac+Mec (i.v)	Mec (i.v)	28	(2), (3), (6), (7)

Abbreviation: T: experimental group; C: control group; Ac: acupuncture; Mec: mecobalamin; p.o: per os; i.m: intramuscular vitamins; i.v: intravenous injection; i.v.drip: intravenous drip; Vit: vitamin. (1) Clinical efficacy; (2) the MCV of the peroneal nerve; (3) the SCV of the peroneal nerve; (4) the MCV of the tibial nerve; (5) the SCV of the tibial nerve; (6) the MCV of the median nerve; (7) the SCV of the median nerve; (8) the MCV of the ulnar nerve; (9) Toronto clinical scoring system (TCSS).

**Table 2:** The characteristics of manual acupuncture.

References	Acupoints	Reinforcing and reducing	Needle retaining time
Fei 2011	Zusanli (ST36), Sanyinjiao (SP6), Pishu (BL20), Shenshu (BL23), Weiwanxiashu (EX-CA)	Mild supplementing and reducing	30 min
Han 2016	Houxi (SI13), Zhaohai (KI6), Zhaohai (KI6), Neiguan (PC6), Waiguan (TE5), Gongsun (SP4), Lieque (LU7), Lieque (LU7)	Mild supplementing and reducing	30 min
Ji 2010	Zusanli (ST36), Sanyinjiao (SP6), Quchi (LI11), Yanglingquan (GB34), Hegu (LI4), Zhongwan (RN12), Xuehai (SP10), Diji (SP8), Yinlingquan (SP9), Fenglong (ST40), Taichong (LR3)	Mild supplementing and reducing	30 min
Li 1998	Zusanli (ST36), Sanyinjiao (SP6), Quchi (LI11), Yanglingquan (GB34), Fenglong (ST40), Taibai (SP3) ⟶ Zutonggu (BL66)	Mild supplementing and reducing	20 min
Li 2005	Zusanli (ST36), Sanyinjiao (SP6), Quchi (LI11), Yanglingquan (GB34), Pishu (BL20), Hegu (LI4), Dazhui (DU14), Weiwanxiashu (EX-CA), Shenshu (BL23), Neiguan (PC6), Xuanzhong (GB39)	Mild supplementing and reducing	20-30 min
Li 2011	Quchi (LI11), Pishu (BL20), Hegu (LI4), Houxi (SI13), Shenshu (BL23), Xiawan (RN10), Zhongwan (RN12), Qihai (RN6), Guanyuan (RN4), Ganshu (BL18)	Mild supplementing and reducing	15 min
Lu 2016	Zusanli (ST36), Pishu (BL20), Shenshu (BL23), Geshu (BL17), Weiwanxiashu (EX-CA), Ganshu (BL18), Taixi (KI3), Ashi point	Mild supplementing and reducing	30 min
Pan 2014	Zusanli (ST36), Sanyinjiao (SP6), Quchi (LI11), Yanglingquan (GB34), Pishu (BL20), Hegu (LI4), Houxi (SI13), Shenshu (BL23), Ganshu (BL18), Guanyuan (RN4), Qihai (RN6), Zhongwan (RN12), Xiawan (RN10), Yinlingquan (SP9), Diji (SP8), Jiexi (ST41), Yongquan (KI1)	Mild supplementing and reducing	20 min
Ren 2007	Zusanli (ST36), Sanyinjiao (SP6), Quchi (LI11), Yanglingquan (GB34), Fengchi (G20), Waiguan (TE5), Weizhong (BL40), Fenglong (ST40), Taichong (LR3), Xuehai (SP10)	Mild supplementing and reducing	20 min
Song 2005	Zusanli (ST36), Sanyinjiao (SP6), Pishu (BL20), Feishu (BL13)	Mild supplementing and reducing	30 min
Wang 2007	Zusanli (ST36), Quchi (LI11), Yanglingquan (GB34), Hegu (LI4), Neiguan (PC6), Yangchi (TE4), Taichong (LR3), Jiexi (ST41)	/	40 min
Wang 2010	Zusanli (ST36), Sanyinjiao (SP6), Quchi (LI11), Yanglingquan (GB34), Jiexi (ST41), Neiting (ST44), Taixi (KI3), Guanyuan (RN4), Geshu (BL17), Ashi point	Mild supplementing and reducing	30 min
Wu 2017	Sanyinjiao (SP6), Taixi (KI3), Weiwanxiashu (EX-CA), Feishu (BL13), Weishu (BL21), Shenshu (BL23)	Mild supplementing and reducing	/
Yan 2007	Zusanli (ST36), Sanyinjiao (SP6), Quchi (LI11), Yanglingquan (GB34), Pishu (BL20), Taichong (LR3), Neiting (ST44), Jiexi (ST41), Xingjian (LR2), Yinlingquan (SP9), Erjian (LI2), Sanjian (LI3), Yangxi (LI5), Yemen (TE2), Zhongzhu (TE3), Qiangu (SI2), Houxi (SI13), Daling (PC7), Weiwanxiashu (EX-CA)	Mild supplementing and reducing	20 min
Yao 2012	Houxi (SI13), Neiguan (PC6), Gongsun (SP4), Waiguan (TE5), Lieque (LU7), Zhaohai (KI6), Lieque (LU7), Zhaohai (KI6)	Mild supplementing and reducing	30 min
Zhao 2007	Zusanli (ST36), Sanyinjiao (SP6), Quchi (LI11), Yanglingquan (GB34), Pishu (BL20), Hegu (LI4), Weiwanxiashu (EX-CA), Shenshu (BL23), Feishu (BL13), Taixi (KI3), Ganshu (BL18)	Mild supplementing and reducing	30 min
Zhao 2016	Houxi (SI13), Zhaohai (KI6), Zhaohai (KI6), Waiguan (TE5), Neiguan (PC6), Lieque (LU7), Gongsun (SP4), Lieque (LU7)	Mild supplementing and reducing	30 min
Zuo 2010	Zusanli (ST36), Sanyinjiao (SP6), Quchi (LI11), Hegu (LI4), Taixi (KI3), Waiguan (TE5), Guanyuan (CV4), Qihai (CV6)	Mild supplementing and reducing	60 min

## Data Availability

We searched against databases and reviewed all literature and then extracted data; additional information was obtained by contacting authors directly via call or email.

## Supplementary Materials

Supplementary Materials Table S1: search strategy. Table S2: PRISMA checklist. Table S3: PubMed search history. Figure S1: the sensitivity analysis of clinical efficacy of acupuncture alone. Figure S2: the sensitivity analysis of the MCV of the peroneal nerve of acupuncture alone. Figure S3: the sensitivity analysis of the SCV of the peroneal nerve of acupuncture alone. Figure S4: the sensitivity analysis of the MCV of the tibial nerve of acupuncture alone. Figure S5: the sensitivity analysis of the SCV of the tibial nerve of acupuncture alone. Figure S6: the sensitivity analysis of the MCV of the median nerve of acupuncture alone. Figure S7: the sensitivity analysis of clinical efficacy of acupuncture combined with vitamin B. Figure S8: the sensitivity analysis of the MCV of the peroneal nerve of acupuncture combined with vitamin B. Figure S9: the sensitivity analysis of the SCV of the peroneal nerve of acupuncture combined with vitamin B. Figure S10: the sensitivity analysis of the MCV of the median nerve of acupuncture combined with vitamin B. Figure S11: the sensitivity analysis of the SCV of the median nerve of acupuncture combined with vitamin B.
