# Supplementary material for: Manual Acupuncture or Combination with Vitamin B to Treat Diabetic Peripheral Neuropathy: A Systematic Review and Meta-Analysis of Randomized Controlled Trials
Source: Biomed Res Int. 2020 Nov 21;2020:4809125. doi: 10.1155/2020/4809125 (PMC8067773; doi:10.1155/2020/4809125)
Supplement: Supplementary Materials — Table S1: search strategy. Table S2: PRISMA checklist. Table S3: PubMed search history. Figure S1: the sensitivity analysis of clinical efficacy of acupuncture alone. Figure S2: the sensitivity analysis of the MCV of the peroneal nerve of acupuncture alone. Figure S3: the sensitivity analysis of the SCV of the peroneal nerve of acupuncture alone. Figure S4: the sensitivity analysis of the MCV of the tibial nerve of acupuncture alone. Figure S5: the sensitivity analysis of the SCV of the tibial nerve of acupuncture alone. Figure S6: the sensitivity analysis of the MCV of the median nerve of acupuncture alone. Figure S7: the sensitivity analysis of clinical efficacy of acupuncture combined with vitamin B. Figure S8: the sensitivity analysis of the MCV of the peroneal nerve of acupuncture combined with vitamin B. Figure S9: the sensitivity analysis of the SCV of the peroneal nerve of acupuncture combined with vitamin B. Figure S10: the sensitivity analysis of the MCV of the median nerve of acupuncture combined with vitamin B. Figure S11: the sensitivity analysis of the SCV of the median nerve of acupuncture combined with vitamin B. [file 4809125.f1.zip › 4809125.f3.docx]

| Search number | Query | Sort By | Filters | Search Details | Results | Time |
| --- | --- | --- | --- | --- | --- | --- |
| 5 | ((acupuncture[Title/Abstract]) OR (Pharmacopuncture[Title/Abstract])) AND (((((((((((((((((((((((((((((((((((((((((((((Diabetic Neuropathy[Title/Abstract]) OR (Neuropathies, Diabetic[Title/Abstract])) OR (Neuropathy, Diabetic[Title/Abstract])) OR (Diabetic Autonomic Neuropathy[Title/Abstract])) OR (Autonomic Neuropathies, Diabetic[Title/Abstract])) OR (Autonomic Neuropathy, Diabetic[Title/Abstract])) OR (Diabetic Autonomic Neuropathies[Title/Abstract])) OR (Neuropathies, Diabetic Autonomic[Title/Abstract])) OR (Neuropathy, Diabetic Autonomic[Title/Abstract])) OR (Diabetic Neuralgia[Title/Abstract])) OR (Diabetic Neuralgias[Title/Abstract])) OR (Neuralgias, Diabetic[Title/Abstract])) OR (Diabetic Neuropathy, Painful[Title/Abstract])) OR (Diabetic Neuropathies, Painful[Title/Abstract])) OR (Neuropathies, Painful Diabetic[Title/Abstract])) OR (Neuropathy, Painful Diabetic[Title/Abstract])) OR (Painful Diabetic Neuropathies[Title/Abstract])) OR (Painful Diabetic Neuropathy[Title/Abstract])) OR (Neuralgia, Diabetic[Title/Abstract])) OR (Symmetric Diabetic Proximal Motor Neuropathy[Title/Abstract])) OR (Asymmetric Diabetic Proximal Motor Neuropathy[Title/Abstract])) OR (Diabetic Asymmetric Polyneuropathy[Title/Abstract])) OR (Asymmetric Polyneuropathies, Diabetic[Title/Abstract])) OR (Asymmetric Polyneuropathy, Diabetic[Title/Abstract])) OR (Diabetic Asymmetric Polyneuropathies[Title/Abstract])) OR (Polyneuropathies, Diabetic Asymmetric[Title/Abstract])) OR (Polyneuropathy, Diabetic Asymmetric[Title/Abstract])) OR (Diabetic Mononeuropathy[Title/Abstract])) OR (Diabetic Mononeuropathies[Title/Abstract])) OR (Mononeuropathies, Diabetic[Title/Abstract])) OR (Mononeuropathy, Diabetic[Title/Abstract])) OR (Diabetic Mononeuropathy Simplex[Title/Abstract])) OR (Diabetic Mononeuropathy Simplices[Title/Abstract])) OR (Mononeuropathy Simplex, Diabetic[Title/Abstract])) OR (Mononeuropathy Simplices, Diabetic[Title/Abstract])) OR (Simplex, Diabetic Mononeuropathy[Title/Abstract])) OR (Simplices, Diabetic Mononeuropathy[Title/Abstract])) OR (Diabetic Amyotrophy[Title/Abstract])) OR (Amyotrophies, Diabetic[Title/Abstract])) OR (Amyotrophy, Diabetic[Title/Abstract])) OR (Diabetic Amyotrophies[Title/Abstract])) OR (Diabetic Polyneuropathy[Title/Abstract])) OR (Diabetic Polyneuropathies[Title/Abstract])) OR (Polyneuropathies, Diabetic[Title/Abstract])) OR (Polyneuropathy, Diabetic[Title/Abstract])) |  |  | ("acupuncture"[Title/Abstract] OR "Pharmacopuncture"[Title/Abstract]) AND (((((((((((((((((((((((((((((((((((((((((((("diabetic neuropathy"[Title/Abstract] OR "neuropathies diabetic"[Title/Abstract]) OR "neuropathy diabetic"[Title/Abstract]) OR "diabetic autonomic neuropathy"[Title/Abstract]) OR "autonomic neuropathies diabetic"[Title/Abstract]) OR "autonomic neuropathy diabetic"[Title/Abstract]) OR "diabetic autonomic neuropathies"[Title/Abstract]) OR "neuropathies diabetic autonomic"[Title/Abstract]) OR "neuropathy diabetic autonomic"[Title/Abstract]) OR "diabetic neuralgia"[Title/Abstract]) OR ((((((((((("diabete"[All Fields] OR "diabetes mellitus"[MeSH Terms]) OR ("diabetes"[All Fields] AND "mellitus"[All Fields])) OR "diabetes mellitus"[All Fields]) OR "diabetes"[All Fields]) OR "diabetes insipidus"[MeSH Terms]) OR ("diabetes"[All Fields] AND "insipidus"[All Fields])) OR "diabetes insipidus"[All Fields]) OR "Diabetic"[All Fields]) OR "diabetics"[All Fields]) OR "diabets"[All Fields]) AND "Neuralgias"[Title/Abstract])) OR ((("Neuralgia"[MeSH Terms] OR "Neuralgia"[All Fields]) OR "Neuralgias"[All Fields]) AND "Diabetic"[Title/Abstract])) OR "diabetic neuropathy painful"[Title/Abstract]) OR "diabetic neuropathies painful"[Title/Abstract]) OR (("Neuropathies"[All Fields] OR "Neuropathy"[All Fields]) AND "painful diabetic"[Title/Abstract])) OR "neuropathy painful diabetic"[Title/Abstract]) OR "painful diabetic neuropathies"[Title/Abstract]) OR "painful diabetic neuropathy"[Title/Abstract]) OR "neuralgia diabetic"[Title/Abstract]) OR ((((((((("symmetric"[All Fields] OR "symmetrical"[All Fields]) OR "symmetrically"[All Fields]) OR "symmetrization"[All Fields]) OR "symmetrize"[All Fields]) OR "symmetrized"[All Fields]) OR "symmetrizes"[All Fields]) OR "symmetrizing"[All Fields]) AND (((((((((("diabete"[All Fields] OR "diabetes mellitus"[MeSH Terms]) OR ("diabetes"[All Fields] AND "mellitus"[All Fields])) OR "diabetes mellitus"[All Fields]) OR "diabetes"[All Fields]) OR "diabetes insipidus"[MeSH Terms]) OR ("diabetes"[All Fields] AND "insipidus"[All Fields])) OR "diabetes insipidus"[All Fields]) OR "Diabetic"[All Fields]) OR "diabetics"[All Fields]) OR "diabets"[All Fields])) AND "proximal motor neuropathy"[Title/Abstract])) OR (((((("Asymmetric"[All Fields] OR "asymmetrical"[All Fields]) OR "asymmetrically"[All Fields]) OR "asymmetrics"[All Fields]) OR "asymmetrization"[All Fields]) AND (((((((((("diabete"[All Fields] OR "diabetes mellitus"[MeSH Terms]) OR ("diabetes"[All Fields] AND "mellitus"[All Fields])) OR "diabetes mellitus"[All Fields]) OR "diabetes"[All Fields]) OR "diabetes insipidus"[MeSH Terms]) OR ("diabetes"[All Fields] AND "insipidus"[All Fields])) OR "diabetes insipidus"[All Fields]) OR "Diabetic"[All Fields]) OR "diabetics"[All Fields]) OR "diabets"[All Fields])) AND "proximal motor neuropathy"[Title/Abstract])) OR ((((((((((("diabete"[All Fields] OR "diabetes mellitus"[MeSH Terms]) OR ("diabetes"[All Fields] AND "mellitus"[All Fields])) OR "diabetes mellitus"[All Fields]) OR "diabetes"[All Fields]) OR "diabetes insipidus"[MeSH Terms]) OR ("diabetes"[All Fields] AND "insipidus"[All Fields])) OR "diabetes insipidus"[All Fields]) OR "Diabetic"[All Fields]) OR "diabetics"[All Fields]) OR "diabets"[All Fields]) AND "asymmetric polyneuropathy"[Title/Abstract])) OR ((((("Asymmetric"[All Fields] OR "asymmetrical"[All Fields]) OR "asymmetrically"[All Fields]) OR "asymmetrics"[All Fields]) OR "asymmetrization"[All Fields]) AND "polyneuropathies diabetic"[Title/Abstract])) OR ((((("Asymmetric"[All Fields] OR "asymmetrical"[All Fields]) OR "asymmetrically"[All Fields]) OR "asymmetrics"[All Fields]) OR "asymmetrization"[All Fields]) AND "polyneuropathy diabetic"[Title/Abstract])) OR (((((((((((("diabete"[All Fields] OR "diabetes mellitus"[MeSH Terms]) OR ("diabetes"[All Fields] AND "mellitus"[All Fields])) OR "diabetes mellitus"[All Fields]) OR "diabetes"[All Fields]) OR "diabetes insipidus"[MeSH Terms]) OR ("diabetes"[All Fields] AND "insipidus"[All Fields])) OR "diabetes insipidus"[All Fields]) OR "Diabetic"[All Fields]) OR "diabetics"[All Fields]) OR "diabets"[All Fields]) AND (((("Asymmetric"[All Fields] OR "asymmetrical"[All Fields]) OR "asymmetrically"[All Fields]) OR "asymmetrics"[All Fields]) OR "asymmetrization"[All Fields])) AND "Polyneuropathies"[Title/Abstract])) OR ((("Polyneuropathies"[MeSH Terms] OR "Polyneuropathies"[All Fields]) OR "Polyneuropathy"[All Fields]) AND "diabetic asymmetric"[Title/Abstract])) OR ((("Polyneuropathies"[MeSH Terms] OR "Polyneuropathies"[All Fields]) OR "Polyneuropathy"[All Fields]) AND "diabetic asymmetric"[Title/Abstract])) OR "diabetic mononeuropathy"[Title/Abstract]) OR "diabetic mononeuropathies"[Title/Abstract]) OR ((("Mononeuropathies"[MeSH Terms] OR "Mononeuropathies"[All Fields]) OR "Mononeuropathy"[All Fields]) AND "Diabetic"[Title/Abstract])) OR "mononeuropathy diabetic"[Title/Abstract]) OR ((((((((((("diabete"[All Fields] OR "diabetes mellitus"[MeSH Terms]) OR ("diabetes"[All Fields] AND "mellitus"[All Fields])) OR "diabetes mellitus"[All Fields]) OR "diabetes"[All Fields]) OR "diabetes insipidus"[MeSH Terms]) OR ("diabetes"[All Fields] AND "insipidus"[All Fields])) OR "diabetes insipidus"[All Fields]) OR "Diabetic"[All Fields]) OR "diabetics"[All Fields]) OR "diabets"[All Fields]) AND "mononeuropathy simplex"[Title/Abstract])) OR ((((("diabetic neuropathies"[MeSH Terms] OR ("Diabetic"[All Fields] AND "Neuropathies"[All Fields])) OR "diabetic neuropathies"[All Fields]) OR ("Diabetic"[All Fields] AND "Mononeuropathy"[All Fields])) OR "diabetic mononeuropathy"[All Fields]) AND "Simplices"[Title/Abstract])) OR ((("Mononeuropathies"[MeSH Terms] OR "Mononeuropathies"[All Fields]) OR "Mononeuropathy"[All Fields]) AND "simplex diabetic"[Title/Abstract])) OR (((("Mononeuropathies"[MeSH Terms] OR "Mononeuropathies"[All Fields]) OR "Mononeuropathy"[All Fields]) AND "Simplices"[All Fields]) AND "Diabetic"[Title/Abstract])) OR (("Simplex"[All Fields] OR "simplexes"[All Fields]) AND "diabetic mononeuropathy"[Title/Abstract])) OR ("Simplices"[All Fields] AND "diabetic mononeuropathy"[Title/Abstract])) OR "diabetic amyotrophy"[Title/Abstract]) OR ((((("muscular atrophy"[MeSH Terms] OR ("muscular"[All Fields] AND "atrophy"[All Fields])) OR "muscular atrophy"[All Fields]) OR "Amyotrophies"[All Fields]) OR "Amyotrophy"[All Fields]) AND "Diabetic"[Title/Abstract])) OR "amyotrophy diabetic"[Title/Abstract]) OR ((((((((((("diabete"[All Fields] OR "diabetes mellitus"[MeSH Terms]) OR ("diabetes"[All Fields] AND "mellitus"[All Fields])) OR "diabetes mellitus"[All Fields]) OR "diabetes"[All Fields]) OR "diabetes insipidus"[MeSH Terms]) OR ("diabetes"[All Fields] AND "insipidus"[All Fields])) OR "diabetes insipidus"[All Fields]) OR "Diabetic"[All Fields]) OR "diabetics"[All Fields]) OR "diabets"[All Fields]) AND "Amyotrophies"[Title/Abstract])) OR "diabetic polyneuropathy"[Title/Abstract]) OR "diabetic polyneuropathies"[Title/Abstract]) OR "polyneuropathies diabetic"[Title/Abstract]) OR "polyneuropathy diabetic"[Title/Abstract]) | 30 | 12:24:13 |
| 4 | (acupuncture[Title/Abstract]) OR (Pharmacopuncture[Title/Abstract]) |  |  | "acupuncture"[Title/Abstract] OR "Pharmacopuncture"[Title/Abstract] | 21,795 | 12:19:55 |
| 3 | ((((((((((((((((((((((((((((((((((((((((((((Diabetic Neuropathy[Title/Abstract]) OR (Neuropathies, Diabetic[Title/Abstract])) OR (Neuropathy, Diabetic[Title/Abstract])) OR (Diabetic Autonomic Neuropathy[Title/Abstract])) OR (Autonomic Neuropathies, Diabetic[Title/Abstract])) OR (Autonomic Neuropathy, Diabetic[Title/Abstract])) OR (Diabetic Autonomic Neuropathies[Title/Abstract])) OR (Neuropathies, Diabetic Autonomic[Title/Abstract])) OR (Neuropathy, Diabetic Autonomic[Title/Abstract])) OR (Diabetic Neuralgia[Title/Abstract])) OR (Diabetic Neuralgias[Title/Abstract])) OR (Neuralgias, Diabetic[Title/Abstract])) OR (Diabetic Neuropathy, Painful[Title/Abstract])) OR (Diabetic Neuropathies, Painful[Title/Abstract])) OR (Neuropathies, Painful Diabetic[Title/Abstract])) OR (Neuropathy, Painful Diabetic[Title/Abstract])) OR (Painful Diabetic Neuropathies[Title/Abstract])) OR (Painful Diabetic Neuropathy[Title/Abstract])) OR (Neuralgia, Diabetic[Title/Abstract])) OR (Symmetric Diabetic Proximal Motor Neuropathy[Title/Abstract])) OR (Asymmetric Diabetic Proximal Motor Neuropathy[Title/Abstract])) OR (Diabetic Asymmetric Polyneuropathy[Title/Abstract])) OR (Asymmetric Polyneuropathies, Diabetic[Title/Abstract])) OR (Asymmetric Polyneuropathy, Diabetic[Title/Abstract])) OR (Diabetic Asymmetric Polyneuropathies[Title/Abstract])) OR (Polyneuropathies, Diabetic Asymmetric[Title/Abstract])) OR (Polyneuropathy, Diabetic Asymmetric[Title/Abstract])) OR (Diabetic Mononeuropathy[Title/Abstract])) OR (Diabetic Mononeuropathies[Title/Abstract])) OR (Mononeuropathies, Diabetic[Title/Abstract])) OR (Mononeuropathy, Diabetic[Title/Abstract])) OR (Diabetic Mononeuropathy Simplex[Title/Abstract])) OR (Diabetic Mononeuropathy Simplices[Title/Abstract])) OR (Mononeuropathy Simplex, Diabetic[Title/Abstract])) OR (Mononeuropathy Simplices, Diabetic[Title/Abstract])) OR (Simplex, Diabetic Mononeuropathy[Title/Abstract])) OR (Simplices, Diabetic Mononeuropathy[Title/Abstract])) OR (Diabetic Amyotrophy[Title/Abstract])) OR (Amyotrophies, Diabetic[Title/Abstract])) OR (Amyotrophy, Diabetic[Title/Abstract])) OR (Diabetic Amyotrophies[Title/Abstract])) OR (Diabetic Polyneuropathy[Title/Abstract])) OR (Diabetic Polyneuropathies[Title/Abstract])) OR (Polyneuropathies, Diabetic[Title/Abstract])) OR (Polyneuropathy, Diabetic[Title/Abstract]) |  |  | ((((((((((((((((((((((((((((((((((((((((((("diabetic neuropathy"[Title/Abstract] OR "neuropathies diabetic"[Title/Abstract]) OR "neuropathy diabetic"[Title/Abstract]) OR "diabetic autonomic neuropathy"[Title/Abstract]) OR "autonomic neuropathies diabetic"[Title/Abstract]) OR "autonomic neuropathy diabetic"[Title/Abstract]) OR "diabetic autonomic neuropathies"[Title/Abstract]) OR "neuropathies diabetic autonomic"[Title/Abstract]) OR "neuropathy diabetic autonomic"[Title/Abstract]) OR "diabetic neuralgia"[Title/Abstract]) OR ((((((((((("diabete"[All Fields] OR "diabetes mellitus"[MeSH Terms]) OR ("diabetes"[All Fields] AND "mellitus"[All Fields])) OR "diabetes mellitus"[All Fields]) OR "diabetes"[All Fields]) OR "diabetes insipidus"[MeSH Terms]) OR ("diabetes"[All Fields] AND "insipidus"[All Fields])) OR "diabetes insipidus"[All Fields]) OR "Diabetic"[All Fields]) OR "diabetics"[All Fields]) OR "diabets"[All Fields]) AND "Neuralgias"[Title/Abstract])) OR ((("Neuralgia"[MeSH Terms] OR "Neuralgia"[All Fields]) OR "Neuralgias"[All Fields]) AND "Diabetic"[Title/Abstract])) OR "diabetic neuropathy painful"[Title/Abstract]) OR "diabetic neuropathies painful"[Title/Abstract]) OR (("Neuropathies"[All Fields] OR "Neuropathy"[All Fields]) AND "painful diabetic"[Title/Abstract])) OR "neuropathy painful diabetic"[Title/Abstract]) OR "painful diabetic neuropathies"[Title/Abstract]) OR "painful diabetic neuropathy"[Title/Abstract]) OR "neuralgia diabetic"[Title/Abstract]) OR ((((((((("symmetric"[All Fields] OR "symmetrical"[All Fields]) OR "symmetrically"[All Fields]) OR "symmetrization"[All Fields]) OR "symmetrize"[All Fields]) OR "symmetrized"[All Fields]) OR "symmetrizes"[All Fields]) OR "symmetrizing"[All Fields]) AND (((((((((("diabete"[All Fields] OR "diabetes mellitus"[MeSH Terms]) OR ("diabetes"[All Fields] AND "mellitus"[All Fields])) OR "diabetes mellitus"[All Fields]) OR "diabetes"[All Fields]) OR "diabetes insipidus"[MeSH Terms]) OR ("diabetes"[All Fields] AND "insipidus"[All Fields])) OR "diabetes insipidus"[All Fields]) OR "Diabetic"[All Fields]) OR "diabetics"[All Fields]) OR "diabets"[All Fields])) AND "proximal motor neuropathy"[Title/Abstract])) OR (((((("Asymmetric"[All Fields] OR "asymmetrical"[All Fields]) OR "asymmetrically"[All Fields]) OR "asymmetrics"[All Fields]) OR "asymmetrization"[All Fields]) AND (((((((((("diabete"[All Fields] OR "diabetes mellitus"[MeSH Terms]) OR ("diabetes"[All Fields] AND "mellitus"[All Fields])) OR "diabetes mellitus"[All Fields]) OR "diabetes"[All Fields]) OR "diabetes insipidus"[MeSH Terms]) OR ("diabetes"[All Fields] AND "insipidus"[All Fields])) OR "diabetes insipidus"[All Fields]) OR "Diabetic"[All Fields]) OR "diabetics"[All Fields]) OR "diabets"[All Fields])) AND "proximal motor neuropathy"[Title/Abstract])) OR ((((((((((("diabete"[All Fields] OR "diabetes mellitus"[MeSH Terms]) OR ("diabetes"[All Fields] AND "mellitus"[All Fields])) OR "diabetes mellitus"[All Fields]) OR "diabetes"[All Fields]) OR "diabetes insipidus"[MeSH Terms]) OR ("diabetes"[All Fields] AND "insipidus"[All Fields])) OR "diabetes insipidus"[All Fields]) OR "Diabetic"[All Fields]) OR "diabetics"[All Fields]) OR "diabets"[All Fields]) AND "asymmetric polyneuropathy"[Title/Abstract])) OR ((((("Asymmetric"[All Fields] OR "asymmetrical"[All Fields]) OR "asymmetrically"[All Fields]) OR "asymmetrics"[All Fields]) OR "asymmetrization"[All Fields]) AND "polyneuropathies diabetic"[Title/Abstract])) OR ((((("Asymmetric"[All Fields] OR "asymmetrical"[All Fields]) OR "asymmetrically"[All Fields]) OR "asymmetrics"[All Fields]) OR "asymmetrization"[All Fields]) AND "polyneuropathy diabetic"[Title/Abstract])) OR (((((((((((("diabete"[All Fields] OR "diabetes mellitus"[MeSH Terms]) OR ("diabetes"[All Fields] AND "mellitus"[All Fields])) OR "diabetes mellitus"[All Fields]) OR "diabetes"[All Fields]) OR "diabetes insipidus"[MeSH Terms]) OR ("diabetes"[All Fields] AND "insipidus"[All Fields])) OR "diabetes insipidus"[All Fields]) OR "Diabetic"[All Fields]) OR "diabetics"[All Fields]) OR "diabets"[All Fields]) AND (((("Asymmetric"[All Fields] OR "asymmetrical"[All Fields]) OR "asymmetrically"[All Fields]) OR "asymmetrics"[All Fields]) OR "asymmetrization"[All Fields])) AND "Polyneuropathies"[Title/Abstract])) OR ((("Polyneuropathies"[MeSH Terms] OR "Polyneuropathies"[All Fields]) OR "Polyneuropathy"[All Fields]) AND "diabetic asymmetric"[Title/Abstract])) OR ((("Polyneuropathies"[MeSH Terms] OR "Polyneuropathies"[All Fields]) OR "Polyneuropathy"[All Fields]) AND "diabetic asymmetric"[Title/Abstract])) OR "diabetic mononeuropathy"[Title/Abstract]) OR "diabetic mononeuropathies"[Title/Abstract]) OR ((("Mononeuropathies"[MeSH Terms] OR "Mononeuropathies"[All Fields]) OR "Mononeuropathy"[All Fields]) AND "Diabetic"[Title/Abstract])) OR "mononeuropathy diabetic"[Title/Abstract]) OR ((((((((((("diabete"[All Fields] OR "diabetes mellitus"[MeSH Terms]) OR ("diabetes"[All Fields] AND "mellitus"[All Fields])) OR "diabetes mellitus"[All Fields]) OR "diabetes"[All Fields]) OR "diabetes insipidus"[MeSH Terms]) OR ("diabetes"[All Fields] AND "insipidus"[All Fields])) OR "diabetes insipidus"[All Fields]) OR "Diabetic"[All Fields]) OR "diabetics"[All Fields]) OR "diabets"[All Fields]) AND "mononeuropathy simplex"[Title/Abstract])) OR ((((("diabetic neuropathies"[MeSH Terms] OR ("Diabetic"[All Fields] AND "Neuropathies"[All Fields])) OR "diabetic neuropathies"[All Fields]) OR ("Diabetic"[All Fields] AND "Mononeuropathy"[All Fields])) OR "diabetic mononeuropathy"[All Fields]) AND "Simplices"[Title/Abstract])) OR ((("Mononeuropathies"[MeSH Terms] OR "Mononeuropathies"[All Fields]) OR "Mononeuropathy"[All Fields]) AND "simplex diabetic"[Title/Abstract])) OR (((("Mononeuropathies"[MeSH Terms] OR "Mononeuropathies"[All Fields]) OR "Mononeuropathy"[All Fields]) AND "Simplices"[All Fields]) AND "Diabetic"[Title/Abstract])) OR (("Simplex"[All Fields] OR "simplexes"[All Fields]) AND "diabetic mononeuropathy"[Title/Abstract])) OR ("Simplices"[All Fields] AND "diabetic mononeuropathy"[Title/Abstract])) OR "diabetic amyotrophy"[Title/Abstract]) OR ((((("muscular atrophy"[MeSH Terms] OR ("muscular"[All Fields] AND "atrophy"[All Fields])) OR "muscular atrophy"[All Fields]) OR "Amyotrophies"[All Fields]) OR "Amyotrophy"[All Fields]) AND "Diabetic"[Title/Abstract])) OR "amyotrophy diabetic"[Title/Abstract]) OR ((((((((((("diabete"[All Fields] OR "diabetes mellitus"[MeSH Terms]) OR ("diabetes"[All Fields] AND "mellitus"[All Fields])) OR "diabetes mellitus"[All Fields]) OR "diabetes"[All Fields]) OR "diabetes insipidus"[MeSH Terms]) OR ("diabetes"[All Fields] AND "insipidus"[All Fields])) OR "diabetes insipidus"[All Fields]) OR "Diabetic"[All Fields]) OR "diabetics"[All Fields]) OR "diabets"[All Fields]) AND "Amyotrophies"[Title/Abstract])) OR "diabetic polyneuropathy"[Title/Abstract]) OR "diabetic polyneuropathies"[Title/Abstract]) OR "polyneuropathies diabetic"[Title/Abstract]) OR "polyneuropathy diabetic"[Title/Abstract] | 9,290 | 12:15:02 |
| 2 | "Acupuncture"[Mesh] |  |  | "Acupuncture"[MeSH Terms] | 1,669 | 11:55:22 |
| 1 | "Diabetic Neuropathies"[Mesh] |  |  | "Diabetic Neuropathies"[MeSH Terms] | 22,248 | 11:53:44 |
