# Supplementary figures and images for: Manual Acupuncture or Combination with Vitamin B to Treat Diabetic Peripheral Neuropathy: A Systematic Review and Meta-Analysis of Randomized Controlled Trials
Source: Biomed Res Int. 2020 Nov 21;2020:4809125. doi: 10.1155/2020/4809125 (PMC8067773; doi:10.1155/2020/4809125)

Meta-analysis estimates, given named study is omitted

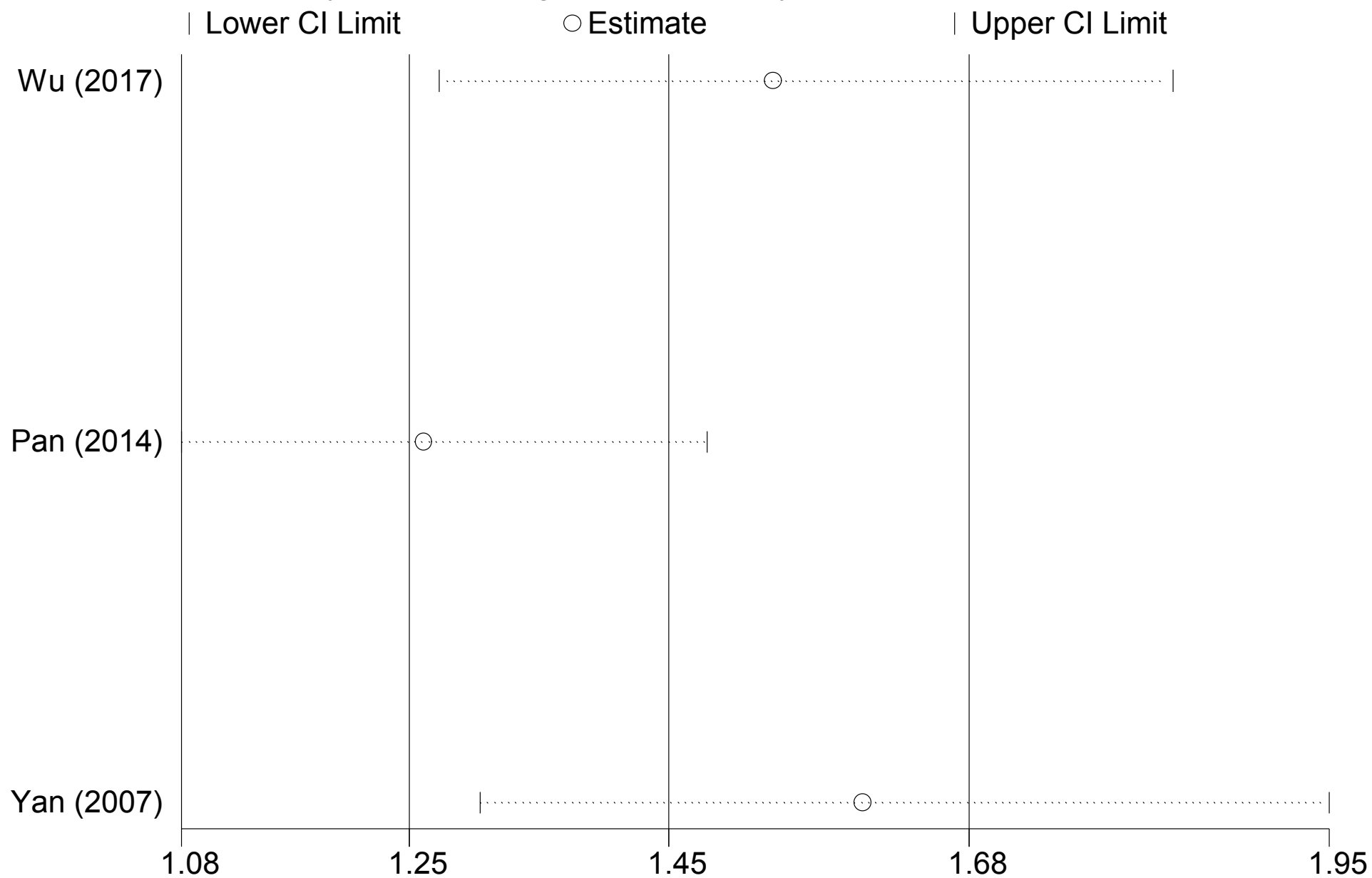

Supplement: Supplementary Materials — Table S1: search strategy. Table S2: PRISMA checklist. Table S3: PubMed search history. Figure S1: the sensitivity analysis of clinical efficacy of acupuncture alone. Figure S2: the sensitivity analysis of the MCV of the peroneal nerve of acupuncture alone. Figure S3: the sensitivity analysis of the SCV of the peroneal nerve of acupuncture alone. Figure S4: the sensitivity analysis of the MCV of the tibial nerve of acupuncture alone. Figure S5: the sensitivity analysis of the SCV of the tibial nerve of acupuncture alone. Figure S6: the sensitivity analysis of the MCV of the median nerve of acupuncture alone. Figure S7: the sensitivity analysis of clinical efficacy of acupuncture combined with vitamin B. Figure S8: the sensitivity analysis of the MCV of the peroneal nerve of acupuncture combined with vitamin B. Figure S9: the sensitivity analysis of the SCV of the peroneal nerve of acupuncture combined with vitamin B. Figure S10: the sensitivity analysis of the MCV of the median nerve of acupuncture combined with vitamin B. Figure S11: the sensitivity analysis of the SCV of the median nerve of acupuncture combined with vitamin B. [file 4809125.f1.zip › 4809125.f10.pdf]

Meta-analysis estimates, given named study is omitted

| Lower CI Limit

○ Estimate

| Upper CI Limit

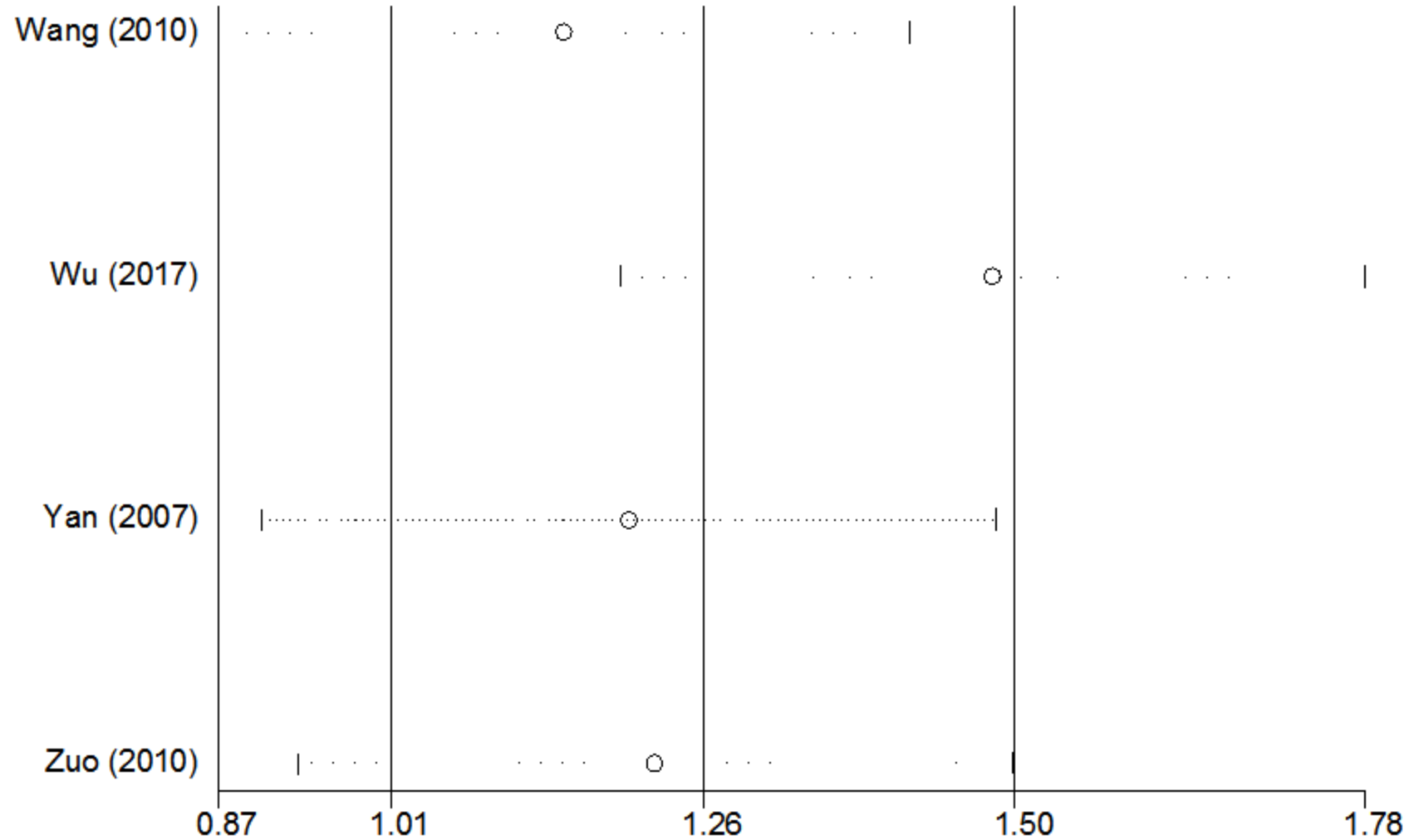

Supplement: Supplementary Materials — Table S1: search strategy. Table S2: PRISMA checklist. Table S3: PubMed search history. Figure S1: the sensitivity analysis of clinical efficacy of acupuncture alone. Figure S2: the sensitivity analysis of the MCV of the peroneal nerve of acupuncture alone. Figure S3: the sensitivity analysis of the SCV of the peroneal nerve of acupuncture alone. Figure S4: the sensitivity analysis of the MCV of the tibial nerve of acupuncture alone. Figure S5: the sensitivity analysis of the SCV of the tibial nerve of acupuncture alone. Figure S6: the sensitivity analysis of the MCV of the median nerve of acupuncture alone. Figure S7: the sensitivity analysis of clinical efficacy of acupuncture combined with vitamin B. Figure S8: the sensitivity analysis of the MCV of the peroneal nerve of acupuncture combined with vitamin B. Figure S9: the sensitivity analysis of the SCV of the peroneal nerve of acupuncture combined with vitamin B. Figure S10: the sensitivity analysis of the MCV of the median nerve of acupuncture combined with vitamin B. Figure S11: the sensitivity analysis of the SCV of the median nerve of acupuncture combined with vitamin B. [file 4809125.f1.zip › 4809125.f11.pdf]

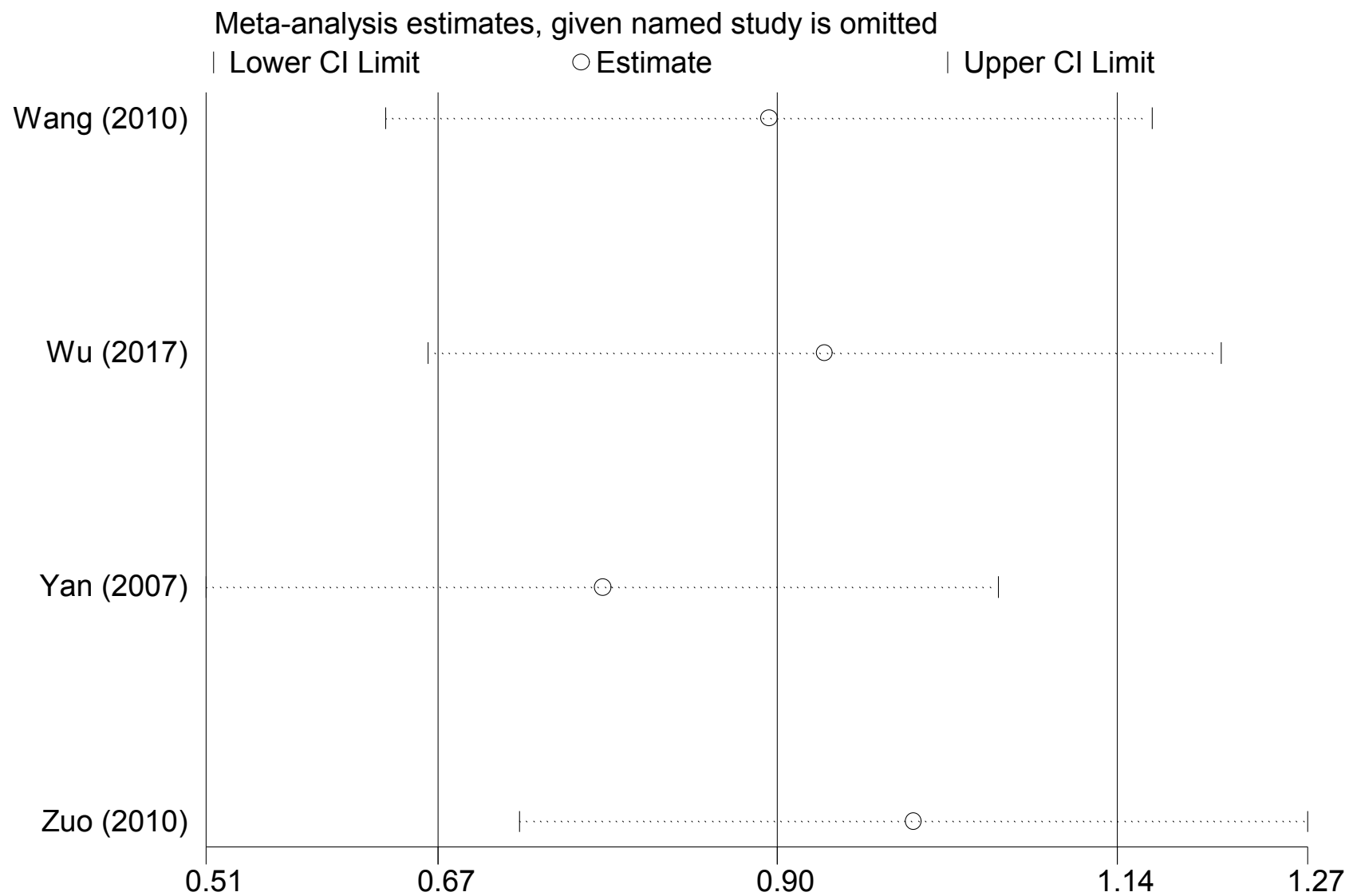

Supplement: Supplementary Materials — Table S1: search strategy. Table S2: PRISMA checklist. Table S3: PubMed search history. Figure S1: the sensitivity analysis of clinical efficacy of acupuncture alone. Figure S2: the sensitivity analysis of the MCV of the peroneal nerve of acupuncture alone. Figure S3: the sensitivity analysis of the SCV of the peroneal nerve of acupuncture alone. Figure S4: the sensitivity analysis of the MCV of the tibial nerve of acupuncture alone. Figure S5: the sensitivity analysis of the SCV of the tibial nerve of acupuncture alone. Figure S6: the sensitivity analysis of the MCV of the median nerve of acupuncture alone. Figure S7: the sensitivity analysis of clinical efficacy of acupuncture combined with vitamin B. Figure S8: the sensitivity analysis of the MCV of the peroneal nerve of acupuncture combined with vitamin B. Figure S9: the sensitivity analysis of the SCV of the peroneal nerve of acupuncture combined with vitamin B. Figure S10: the sensitivity analysis of the MCV of the median nerve of acupuncture combined with vitamin B. Figure S11: the sensitivity analysis of the SCV of the median nerve of acupuncture combined with vitamin B. [file 4809125.f1.zip › 4809125.f12.pdf]

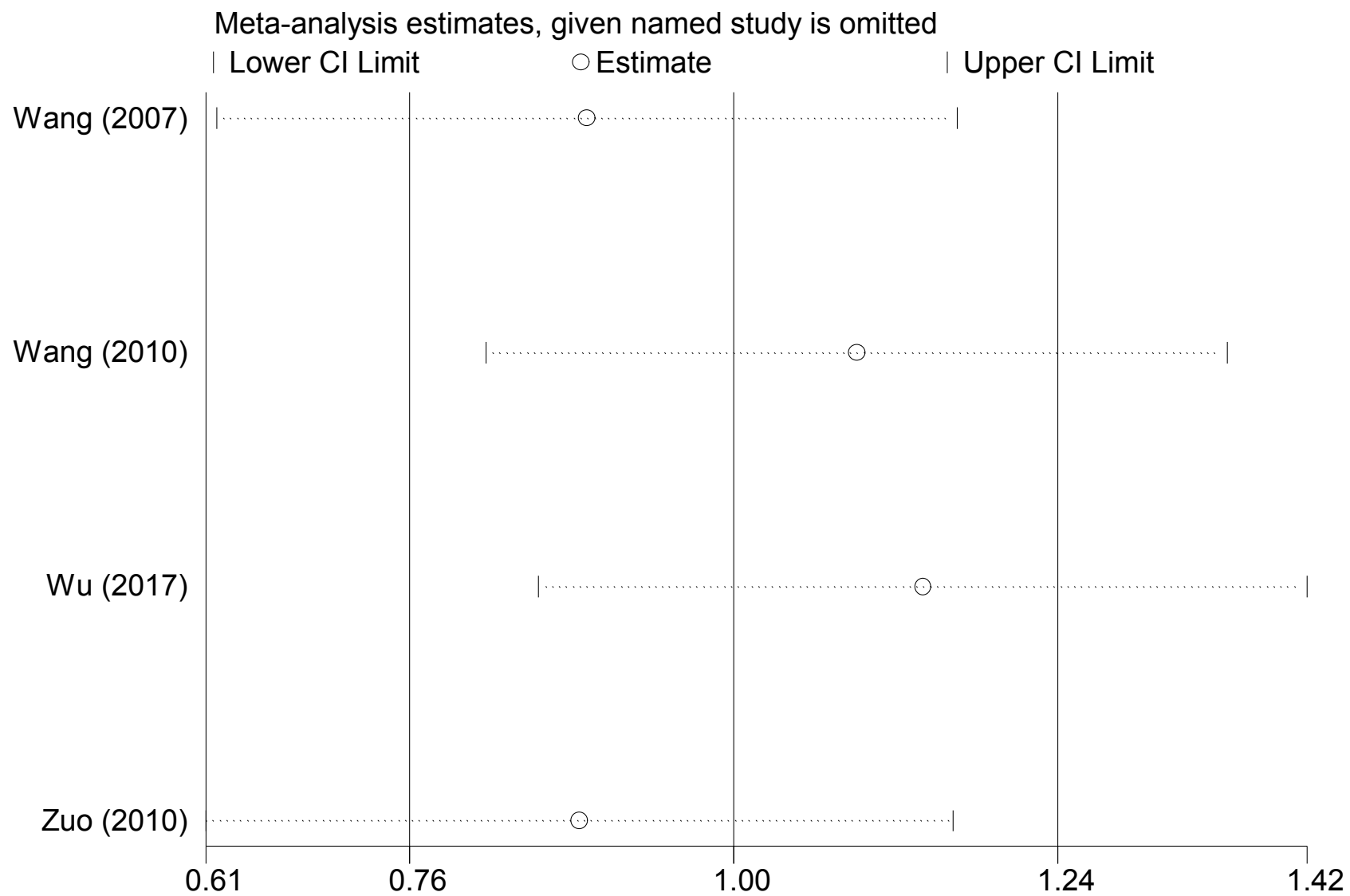

Supplement: Supplementary Materials — Table S1: search strategy. Table S2: PRISMA checklist. Table S3: PubMed search history. Figure S1: the sensitivity analysis of clinical efficacy of acupuncture alone. Figure S2: the sensitivity analysis of the MCV of the peroneal nerve of acupuncture alone. Figure S3: the sensitivity analysis of the SCV of the peroneal nerve of acupuncture alone. Figure S4: the sensitivity analysis of the MCV of the tibial nerve of acupuncture alone. Figure S5: the sensitivity analysis of the SCV of the tibial nerve of acupuncture alone. Figure S6: the sensitivity analysis of the MCV of the median nerve of acupuncture alone. Figure S7: the sensitivity analysis of clinical efficacy of acupuncture combined with vitamin B. Figure S8: the sensitivity analysis of the MCV of the peroneal nerve of acupuncture combined with vitamin B. Figure S9: the sensitivity analysis of the SCV of the peroneal nerve of acupuncture combined with vitamin B. Figure S10: the sensitivity analysis of the MCV of the median nerve of acupuncture combined with vitamin B. Figure S11: the sensitivity analysis of the SCV of the median nerve of acupuncture combined with vitamin B. [file 4809125.f1.zip › 4809125.f13.pdf]

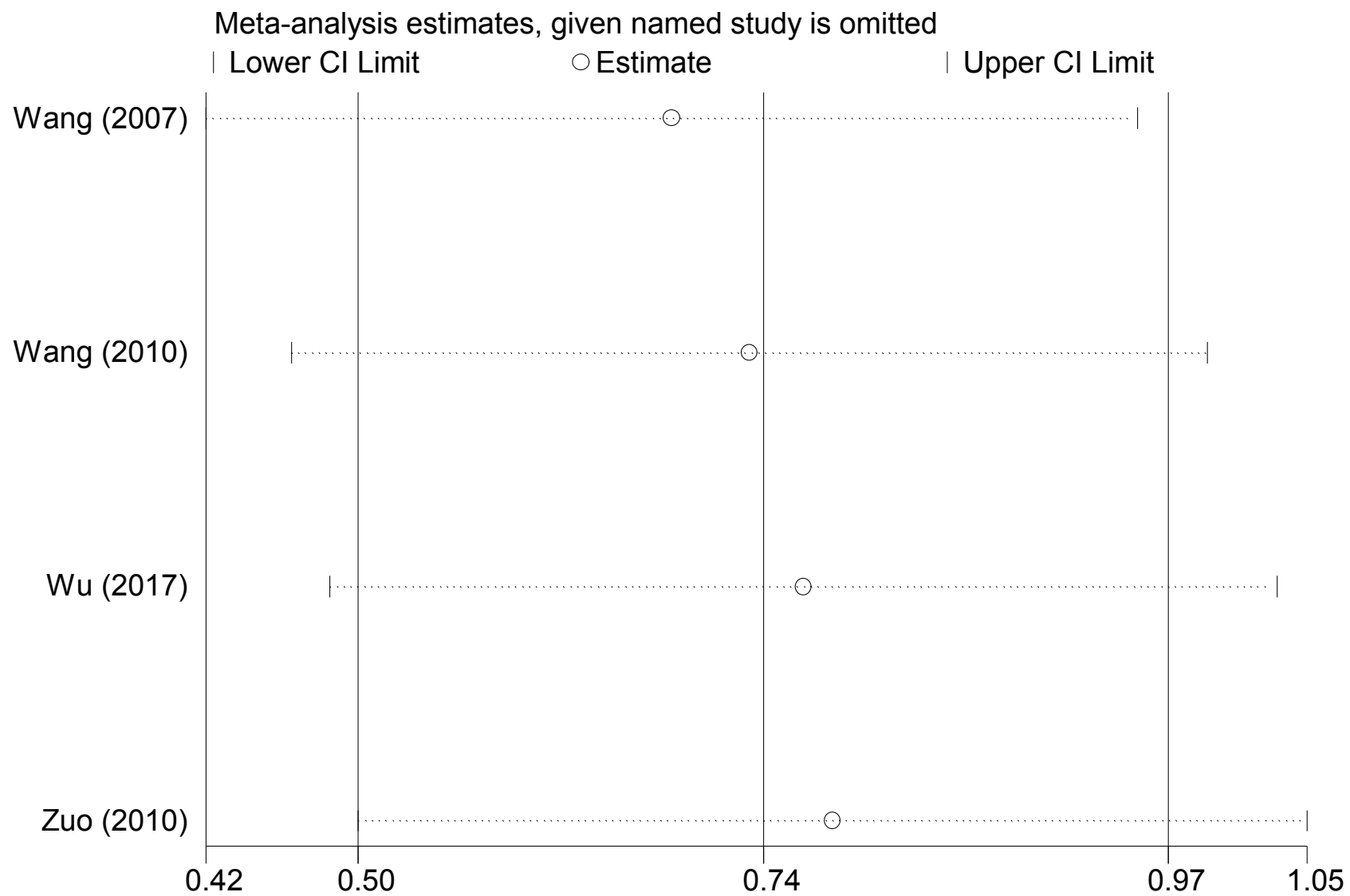

Supplement: Supplementary Materials — Table S1: search strategy. Table S2: PRISMA checklist. Table S3: PubMed search history. Figure S1: the sensitivity analysis of clinical efficacy of acupuncture alone. Figure S2: the sensitivity analysis of the MCV of the peroneal nerve of acupuncture alone. Figure S3: the sensitivity analysis of the SCV of the peroneal nerve of acupuncture alone. Figure S4: the sensitivity analysis of the MCV of the tibial nerve of acupuncture alone. Figure S5: the sensitivity analysis of the SCV of the tibial nerve of acupuncture alone. Figure S6: the sensitivity analysis of the MCV of the median nerve of acupuncture alone. Figure S7: the sensitivity analysis of clinical efficacy of acupuncture combined with vitamin B. Figure S8: the sensitivity analysis of the MCV of the peroneal nerve of acupuncture combined with vitamin B. Figure S9: the sensitivity analysis of the SCV of the peroneal nerve of acupuncture combined with vitamin B. Figure S10: the sensitivity analysis of the MCV of the median nerve of acupuncture combined with vitamin B. Figure S11: the sensitivity analysis of the SCV of the median nerve of acupuncture combined with vitamin B. [file 4809125.f1.zip › 4809125.f14.pdf]

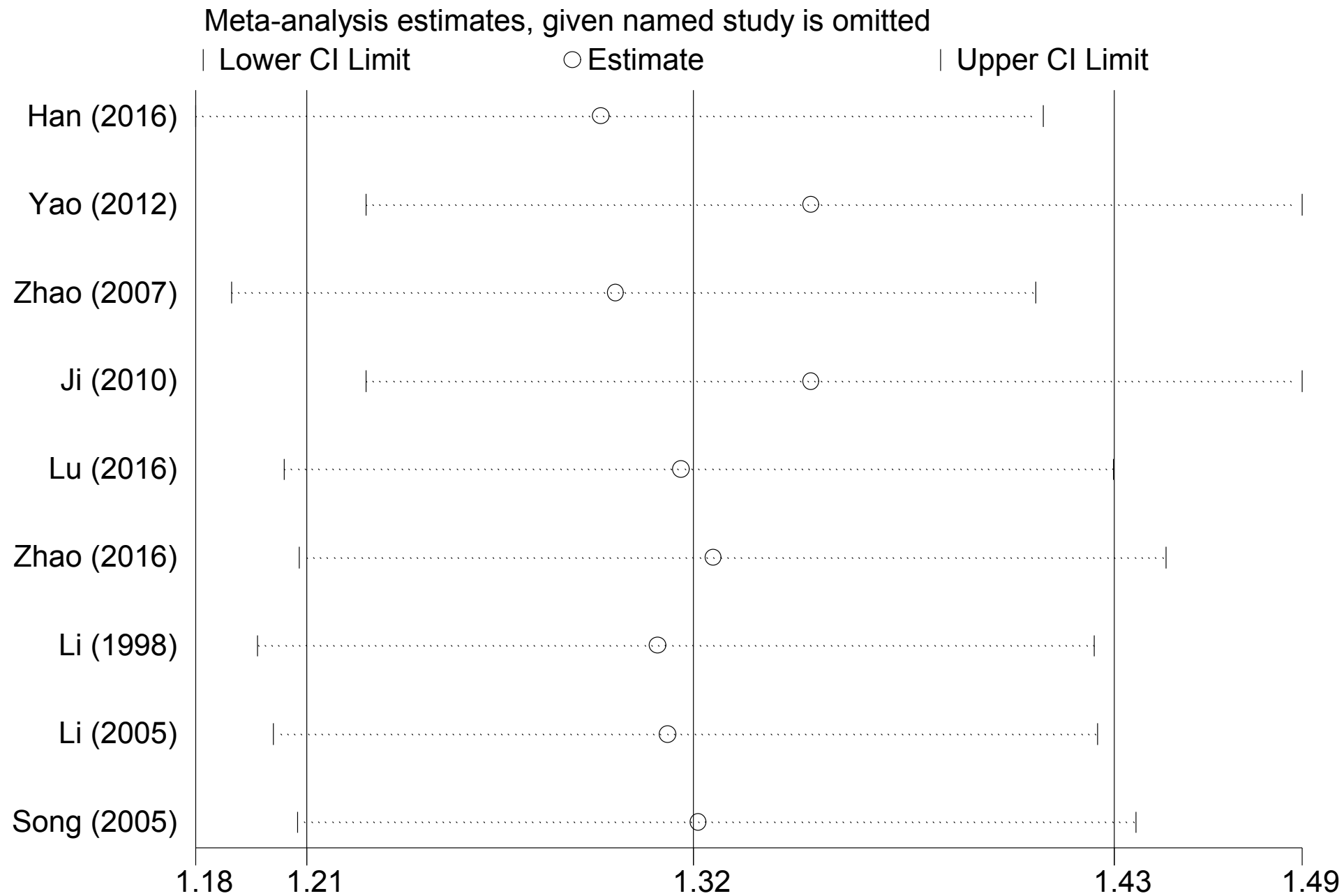

Supplement: Supplementary Materials — Table S1: search strategy. Table S2: PRISMA checklist. Table S3: PubMed search history. Figure S1: the sensitivity analysis of clinical efficacy of acupuncture alone. Figure S2: the sensitivity analysis of the MCV of the peroneal nerve of acupuncture alone. Figure S3: the sensitivity analysis of the SCV of the peroneal nerve of acupuncture alone. Figure S4: the sensitivity analysis of the MCV of the tibial nerve of acupuncture alone. Figure S5: the sensitivity analysis of the SCV of the tibial nerve of acupuncture alone. Figure S6: the sensitivity analysis of the MCV of the median nerve of acupuncture alone. Figure S7: the sensitivity analysis of clinical efficacy of acupuncture combined with vitamin B. Figure S8: the sensitivity analysis of the MCV of the peroneal nerve of acupuncture combined with vitamin B. Figure S9: the sensitivity analysis of the SCV of the peroneal nerve of acupuncture combined with vitamin B. Figure S10: the sensitivity analysis of the MCV of the median nerve of acupuncture combined with vitamin B. Figure S11: the sensitivity analysis of the SCV of the median nerve of acupuncture combined with vitamin B. [file 4809125.f1.zip › 4809125.f4.pdf]

Meta-analysis estimates, given named study is omitted

| Lower CI Limit

○ Estimate

| Upper CI Limit

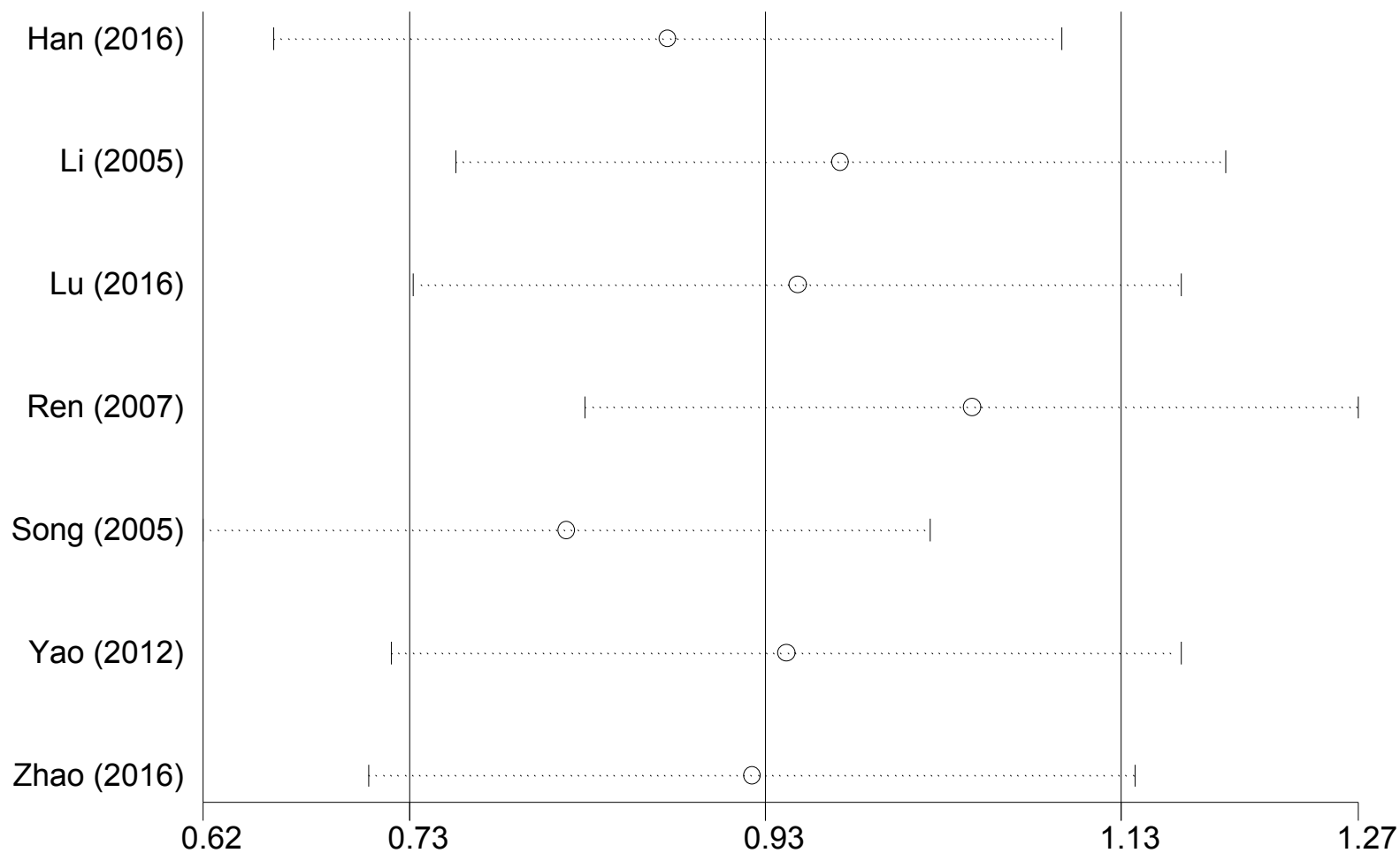

Supplement: Supplementary Materials — Table S1: search strategy. Table S2: PRISMA checklist. Table S3: PubMed search history. Figure S1: the sensitivity analysis of clinical efficacy of acupuncture alone. Figure S2: the sensitivity analysis of the MCV of the peroneal nerve of acupuncture alone. Figure S3: the sensitivity analysis of the SCV of the peroneal nerve of acupuncture alone. Figure S4: the sensitivity analysis of the MCV of the tibial nerve of acupuncture alone. Figure S5: the sensitivity analysis of the SCV of the tibial nerve of acupuncture alone. Figure S6: the sensitivity analysis of the MCV of the median nerve of acupuncture alone. Figure S7: the sensitivity analysis of clinical efficacy of acupuncture combined with vitamin B. Figure S8: the sensitivity analysis of the MCV of the peroneal nerve of acupuncture combined with vitamin B. Figure S9: the sensitivity analysis of the SCV of the peroneal nerve of acupuncture combined with vitamin B. Figure S10: the sensitivity analysis of the MCV of the median nerve of acupuncture combined with vitamin B. Figure S11: the sensitivity analysis of the SCV of the median nerve of acupuncture combined with vitamin B. [file 4809125.f1.zip › 4809125.f5.pdf]

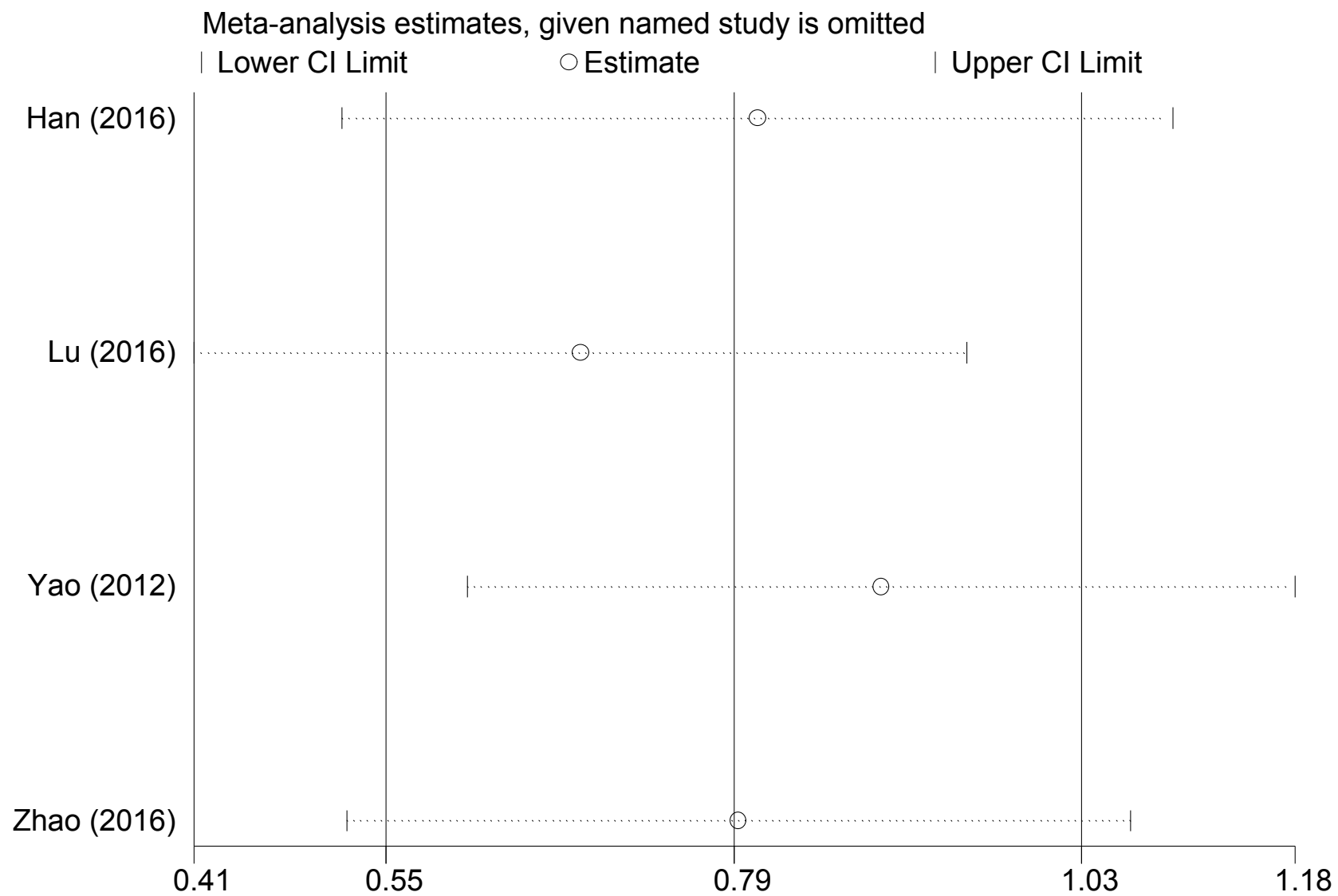

Supplement: Supplementary Materials — Table S1: search strategy. Table S2: PRISMA checklist. Table S3: PubMed search history. Figure S1: the sensitivity analysis of clinical efficacy of acupuncture alone. Figure S2: the sensitivity analysis of the MCV of the peroneal nerve of acupuncture alone. Figure S3: the sensitivity analysis of the SCV of the peroneal nerve of acupuncture alone. Figure S4: the sensitivity analysis of the MCV of the tibial nerve of acupuncture alone. Figure S5: the sensitivity analysis of the SCV of the tibial nerve of acupuncture alone. Figure S6: the sensitivity analysis of the MCV of the median nerve of acupuncture alone. Figure S7: the sensitivity analysis of clinical efficacy of acupuncture combined with vitamin B. Figure S8: the sensitivity analysis of the MCV of the peroneal nerve of acupuncture combined with vitamin B. Figure S9: the sensitivity analysis of the SCV of the peroneal nerve of acupuncture combined with vitamin B. Figure S10: the sensitivity analysis of the MCV of the median nerve of acupuncture combined with vitamin B. Figure S11: the sensitivity analysis of the SCV of the median nerve of acupuncture combined with vitamin B. [file 4809125.f1.zip › 4809125.f6.pdf]

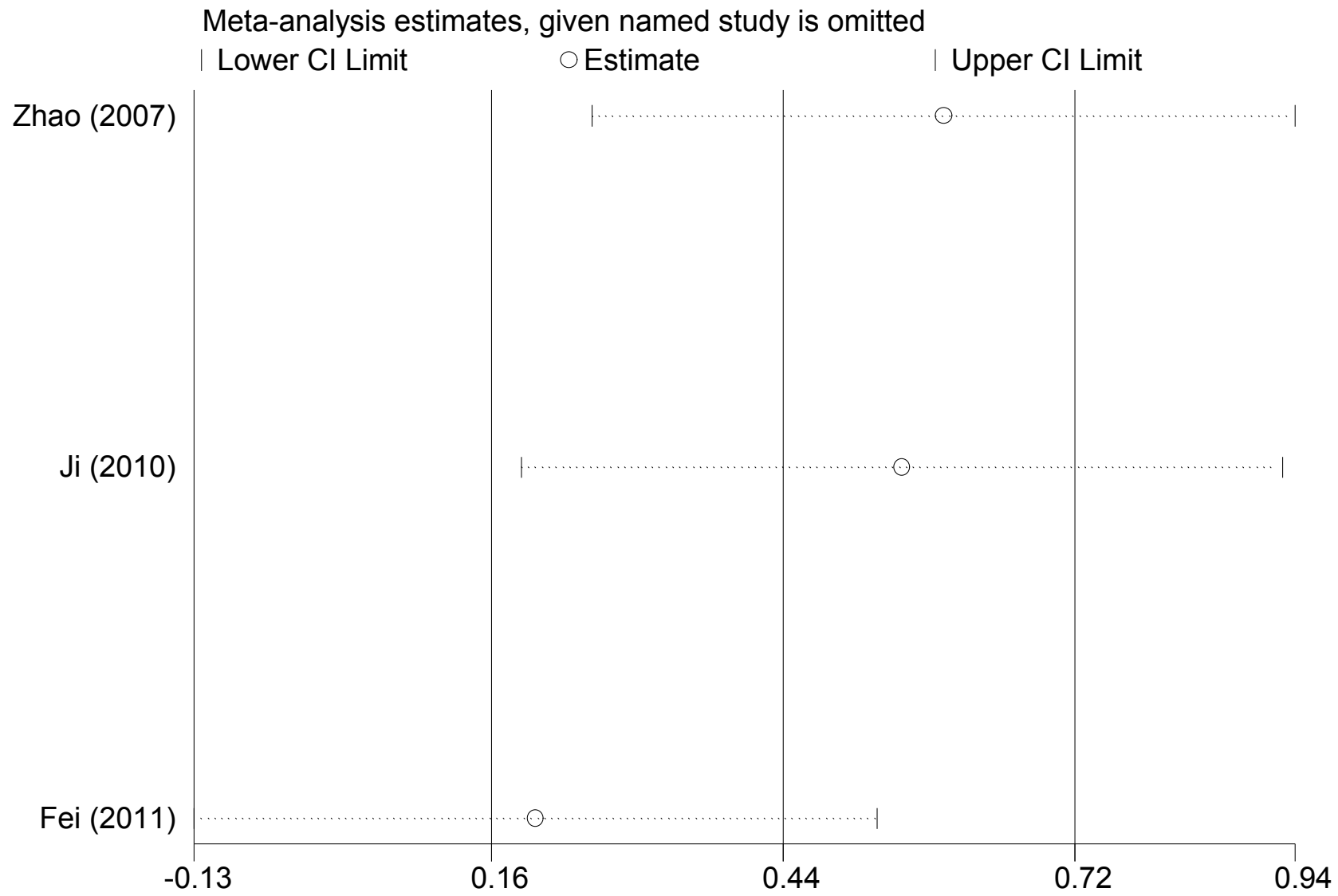

Supplement: Supplementary Materials — Table S1: search strategy. Table S2: PRISMA checklist. Table S3: PubMed search history. Figure S1: the sensitivity analysis of clinical efficacy of acupuncture alone. Figure S2: the sensitivity analysis of the MCV of the peroneal nerve of acupuncture alone. Figure S3: the sensitivity analysis of the SCV of the peroneal nerve of acupuncture alone. Figure S4: the sensitivity analysis of the MCV of the tibial nerve of acupuncture alone. Figure S5: the sensitivity analysis of the SCV of the tibial nerve of acupuncture alone. Figure S6: the sensitivity analysis of the MCV of the median nerve of acupuncture alone. Figure S7: the sensitivity analysis of clinical efficacy of acupuncture combined with vitamin B. Figure S8: the sensitivity analysis of the MCV of the peroneal nerve of acupuncture combined with vitamin B. Figure S9: the sensitivity analysis of the SCV of the peroneal nerve of acupuncture combined with vitamin B. Figure S10: the sensitivity analysis of the MCV of the median nerve of acupuncture combined with vitamin B. Figure S11: the sensitivity analysis of the SCV of the median nerve of acupuncture combined with vitamin B. [file 4809125.f1.zip › 4809125.f7.pdf]

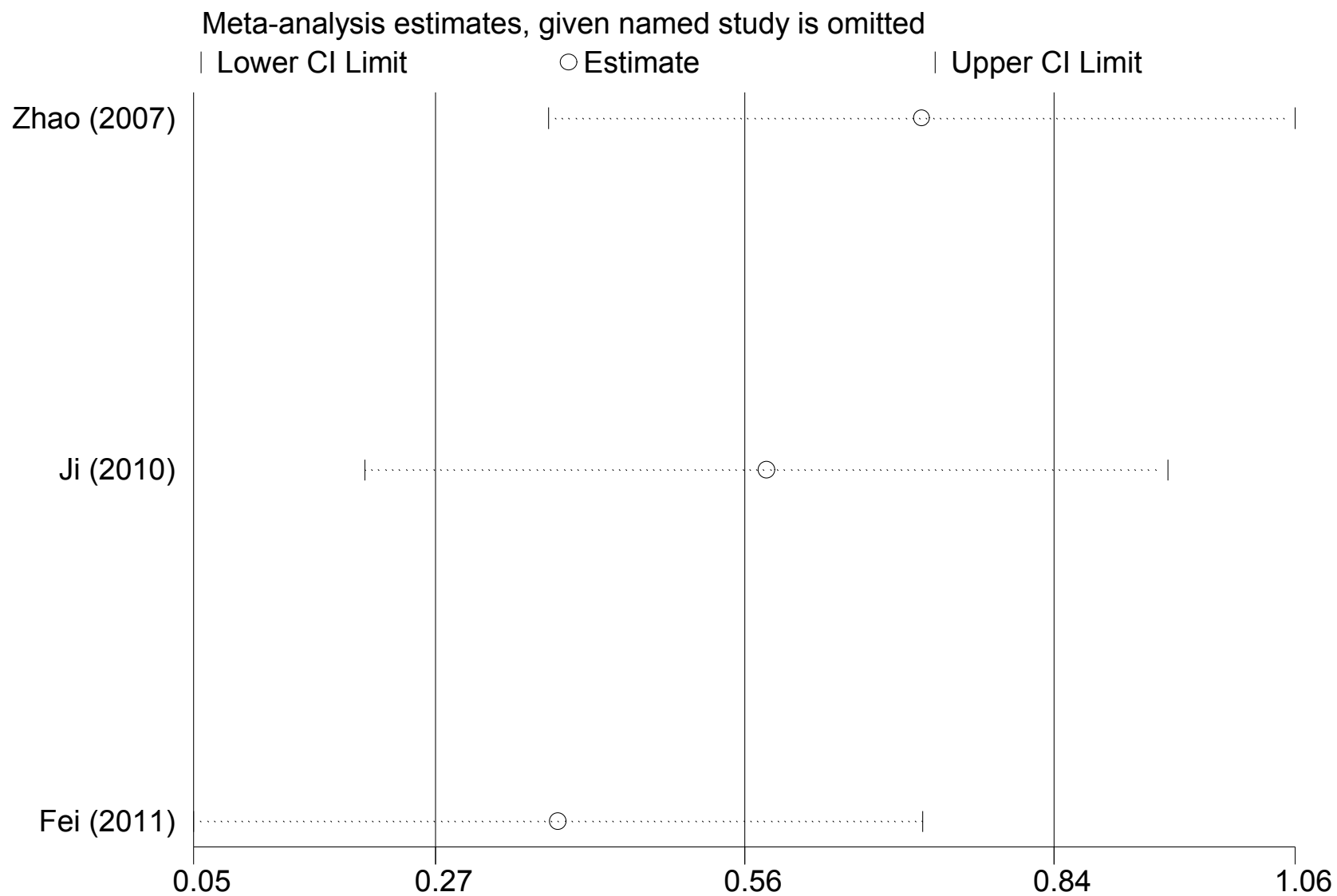

Supplement: Supplementary Materials — Table S1: search strategy. Table S2: PRISMA checklist. Table S3: PubMed search history. Figure S1: the sensitivity analysis of clinical efficacy of acupuncture alone. Figure S2: the sensitivity analysis of the MCV of the peroneal nerve of acupuncture alone. Figure S3: the sensitivity analysis of the SCV of the peroneal nerve of acupuncture alone. Figure S4: the sensitivity analysis of the MCV of the tibial nerve of acupuncture alone. Figure S5: the sensitivity analysis of the SCV of the tibial nerve of acupuncture alone. Figure S6: the sensitivity analysis of the MCV of the median nerve of acupuncture alone. Figure S7: the sensitivity analysis of clinical efficacy of acupuncture combined with vitamin B. Figure S8: the sensitivity analysis of the MCV of the peroneal nerve of acupuncture combined with vitamin B. Figure S9: the sensitivity analysis of the SCV of the peroneal nerve of acupuncture combined with vitamin B. Figure S10: the sensitivity analysis of the MCV of the median nerve of acupuncture combined with vitamin B. Figure S11: the sensitivity analysis of the SCV of the median nerve of acupuncture combined with vitamin B. [file 4809125.f1.zip › 4809125.f8.pdf]

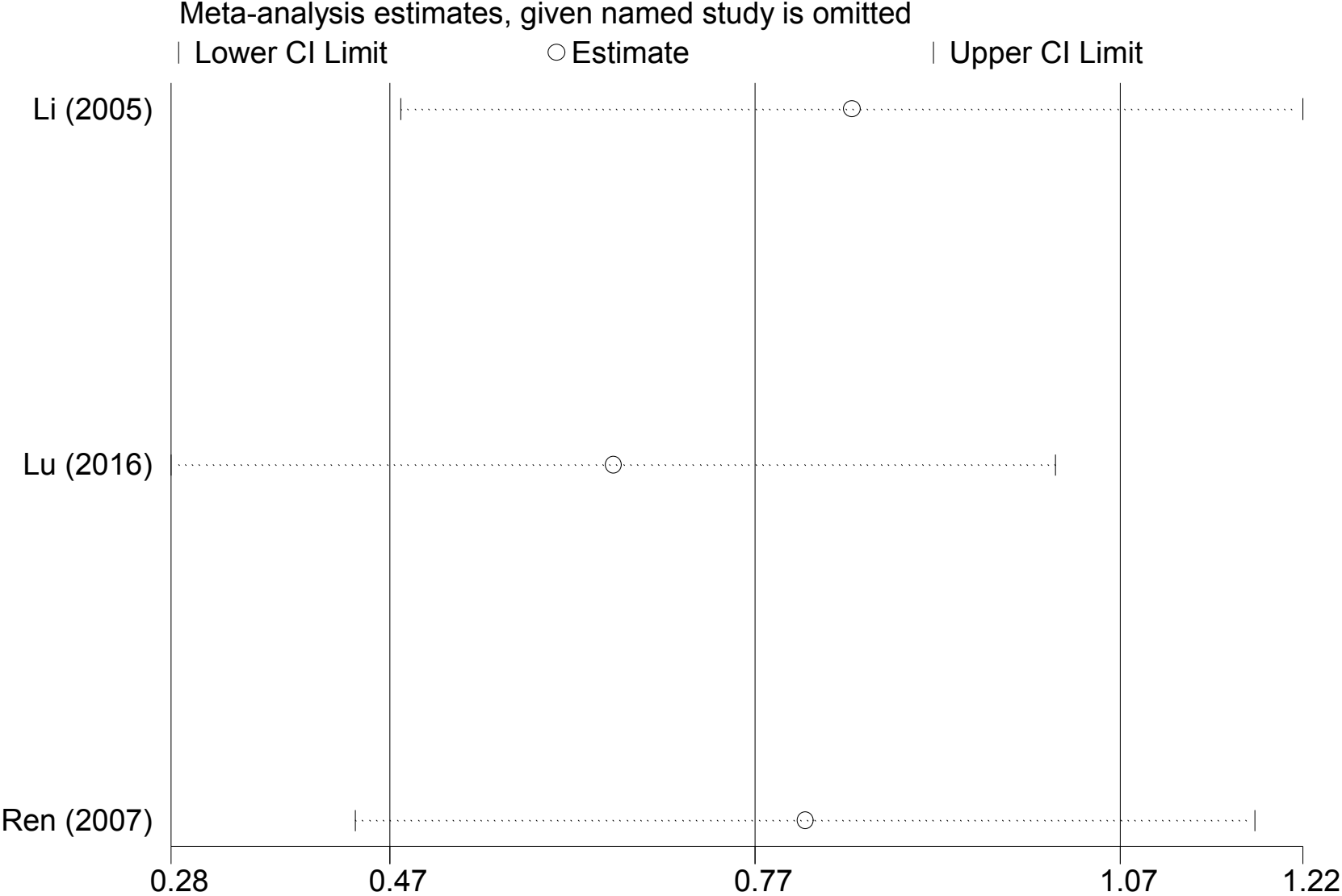

Supplement: Supplementary Materials — Table S1: search strategy. Table S2: PRISMA checklist. Table S3: PubMed search history. Figure S1: the sensitivity analysis of clinical efficacy of acupuncture alone. Figure S2: the sensitivity analysis of the MCV of the peroneal nerve of acupuncture alone. Figure S3: the sensitivity analysis of the SCV of the peroneal nerve of acupuncture alone. Figure S4: the sensitivity analysis of the MCV of the tibial nerve of acupuncture alone. Figure S5: the sensitivity analysis of the SCV of the tibial nerve of acupuncture alone. Figure S6: the sensitivity analysis of the MCV of the median nerve of acupuncture alone. Figure S7: the sensitivity analysis of clinical efficacy of acupuncture combined with vitamin B. Figure S8: the sensitivity analysis of the MCV of the peroneal nerve of acupuncture combined with vitamin B. Figure S9: the sensitivity analysis of the SCV of the peroneal nerve of acupuncture combined with vitamin B. Figure S10: the sensitivity analysis of the MCV of the median nerve of acupuncture combined with vitamin B. Figure S11: the sensitivity analysis of the SCV of the median nerve of acupuncture combined with vitamin B. [file 4809125.f1.zip › 4809125.f9.pdf]
