# Supplementary material for: Manual Acupuncture or Combination with Vitamin B to Treat Diabetic Peripheral Neuropathy: A Systematic Review and Meta-Analysis of Randomized Controlled Trials
Source: Biomed Res Int. 2020 Nov 21;2020:4809125. doi: 10.1155/2020/4809125 (PMC8067773; doi:10.1155/2020/4809125)
Supplement: Supplementary Materials — Table S1: search strategy. Table S2: PRISMA checklist. Table S3: PubMed search history. Figure S1: the sensitivity analysis of clinical efficacy of acupuncture alone. Figure S2: the sensitivity analysis of the MCV of the peroneal nerve of acupuncture alone. Figure S3: the sensitivity analysis of the SCV of the peroneal nerve of acupuncture alone. Figure S4: the sensitivity analysis of the MCV of the tibial nerve of acupuncture alone. Figure S5: the sensitivity analysis of the SCV of the tibial nerve of acupuncture alone. Figure S6: the sensitivity analysis of the MCV of the median nerve of acupuncture alone. Figure S7: the sensitivity analysis of clinical efficacy of acupuncture combined with vitamin B. Figure S8: the sensitivity analysis of the MCV of the peroneal nerve of acupuncture combined with vitamin B. Figure S9: the sensitivity analysis of the SCV of the peroneal nerve of acupuncture combined with vitamin B. Figure S10: the sensitivity analysis of the MCV of the median nerve of acupuncture combined with vitamin B. Figure S11: the sensitivity analysis of the SCV of the median nerve of acupuncture combined with vitamin B. [file 4809125.f1.zip › 4809125.f1.doc]

Embase

Session Results

.......................................................

No. Query Results	Results Date

#3. #1 AND #2	64 5 Apr 2020

#2. acupuncture:ti,ab,kw	31,556 5 Apr 2020

#1. 'diabetic neuropathy':ti,ab,kw OR dpn:ti,ab,kw OR	15,368 5 Apr 2020

'neuropathies, diabetic':ti,ab,kw OR

'neuropathy, diabetic':ti,ab,kw OR 'autonomic

neuropathies, diabetic':ti,ab,kw OR

'diabetic autonomic neuropathy':ti,ab,kw OR

'diabetic autonomic neuropathies':ti,ab,kw OR

'neuropathies, diabetic autonomic':ti,ab,kw OR

'neuropathy, diabetic autonomic':ti,ab,kw OR

'diabetic neuralgia':ti,ab,kw OR

'diabetic neuralgias':ti,ab,kw OR

'neuralgias, diabetic':ti,ab,kw OR

'diabetic neuropathy, painful':ti,ab,kw OR

'diabetic neuropathies, painful':ti,ab,kw OR

'neuropathies, painful diabetic':ti,ab,kw OR

'neuropathy, painful diabetic':ti,ab,kw OR

'painful diabetic neuropathies':ti,ab,kw OR

'painful diabetic neuropathy':ti,ab,kw OR

'neuralgia, diabetic':ti,ab,kw OR

'symmetric diabetic proximal motor

neuropathy':ti,ab,kw OR

'asymmetric diabetic proximal motor

neuropathy':ti,ab,kw OR 'diabetic asymmetric

polyneuropathy':ti,ab,kw OR 'asymmetric

polyneuropathies, diabetic':ti,ab,kw OR

'asymmetric polyneuropathy, diabetic':ti,ab,kw OR

'diabetic asymmetric polyneuropathies':ti,ab,kw

OR 'polyneuropathy, diabetic asymmetric':ti,ab,kw

OR

'polyneuropathies, diabetic asymmetric':ti,ab,kw

OR 'diabetic mononeuropathy':ti,ab,kw OR

'diabetic mononeuropathies':ti,ab,kw OR

'mononeuropathies, diabetic':ti,ab,kw OR

'mononeuropathy, diabetic':ti,ab,kw OR

'diabetic mononeuropathy simplex':ti,ab,kw OR

'diabetic mononeuropathy simplices':ti,ab,kw OR

'mononeuropathy simplex, diabetic':ti,ab,kw OR

'mononeuropathy simplices, diabetic':ti,ab,kw OR

'simplex, diabetic mononeuropathy':ti,ab,kw OR

'simplices, diabetic mononeuropathy':ti,ab,kw OR

'diabetic amyotrophy':ti,ab,kw OR

'amyotrophies, diabetic':ti,ab,kw OR

'amyotrophy, diabetic':ti,ab,kw OR

'diabetic amyotrophies':ti,ab,kw OR

'diabetic polyneuropathy':ti,ab,kw OR

'diabetic polyneuropathies':ti,ab,kw OR

'polyneuropathies, diabetic':ti,ab,kw OR

'polyneuropathy, diabetic':ti,ab,kw

The search formula of CNKI

(SU = '糖尿病周围神经病变'OR SU = '糖尿病周围神经病'OR SU = '糖尿病末梢神经病变'OR SU = '糖尿病性周围神经病变'OR SU = '糖尿病性周围神经病'OR SU = '糖尿病外周神经病变'OR SU = '糖尿病周围神经性病变'OR SU = '糖尿病周围病变'OR SU = '糖尿病周围神经病' OR SU = 'DPN' OR SU = 'diabetes peripheral neuropathy' OR SU = 'diabetic peripheral neuropathy' OR SU = 'Diabetes Polyneuropathy ' OR SU = 'diabetic neuropathy' OR SU = 'diabetic peripheral nerve disease' OR SU = 'diabetic peripheral nerve lesion' OR SU = 'diabetic peripheral neuropathies' OR SU = 'diabetic peripheral neuropathy' ) AND (SU = '针' ) AND (FT = '随机' OR FT = '对照' OR FT= '临床' OR FT = '患者')
